# Control of *Listeria monocytogenes* infection requires classical IL-6 signaling in myeloid cells

**DOI:** 10.1371/journal.pone.0203395

**Published:** 2018-08-31

**Authors:** Karsten Lücke, Isabell Yan, Sonja Krohn, Annika Volmari, Stefanie Klinge, Joanna Schmid, Valéa Schumacher, Oliver M. Steinmetz, Stefan Rose-John, Hans-Willi Mittrücker

**Affiliations:** 1 Institute of Immunology, University Medical Centre Hamburg-Eppendorf, Hamburg, Germany; 2 III. Medical Clinic and Polyclinic, University Medical Centre Hamburg-Eppendorf, Hamburg, Germany; 3 I. Medical Clinic and Polyclinic, University Medical Centre Hamburg-Eppendorf, Hamburg, Germany; 4 Institute for Biochemistry, Medical Faculty, Christian Albrechts University, Kiel, Germany; Karolinska Institutet, SWEDEN

## Abstract

IL-6 is required for the response of mice against *Listeria monocytogenes*. Control of infection depends on classical IL-6 signaling via membrane IL-6Rα, but IL-6 target cells and protective mechanisms remain unclear. We used mice with IL-6Rα-deficiency in T cells (*Il6ra*^fl/fl^×CD4^cre^) or myeloid cells (*Il6ra*^fl/fl^×LysM^cre^) to define the role of these cells in IL-6-mediated protection. Abrogation of IL-6Rα in T cells did not interfere with bacteria control and induction of T_H_1 and CD8^+^ T-cell responses. IL-6Rα-deficiency in myeloid cells caused significant defects in listeria control. This defect was not associated with reduced recruitment of granulocytes and inflammatory monocytes, and both cell populations were activated and not impaired in cytokine production. However, IL-6Rα-deficient inflammatory monocytes displayed diminished expression of IL-4Rα and of CD38, a protein required for phagocytosis and innate control of listeria. *In vitro* studies revealed that IL-4 and IL-6 cooperated in induction of CD38. In listeria-infected mice, phagocytic activity of inflammatory monocytes correlated with CD38 expression levels on cells and inflammatory monocytes of *Il6ra*^fl/fl^×LysM^cre^ mice were significantly impaired in phagocytosis. In conclusion, we demonstrate that inhibition of classical IL-6 signaling in myeloid cells causes alterations in differentiation and function of these cells, which subsequently prevent effective control of *L*. *monocytogenes*.

## Introduction

The cytokine IL-6 has both pro- and anti-inflammatory activities in the immune system [1]. IL-6 controls neutrophil maturation and recruitment to sites of infection [2]. In monocytes, IL-6 regulates differentiation through upregulation of macrophage colony-stimulating factor receptor (M-CSFR) [3]. It also induces IL-4Rα expression and supports formation of alternatively activated macrophages [4, 5]. Furthermore, IL-6 is a central driver of the acute phase response. IL-6 is likewise involved in the adaptive response. IL-6 supports the production of antibodies in B cells either directly or by inducing formation of T follicular helper (T_FH_) cells [6, 7]. Differentiation of naive CD4^+^ T cells to T_H_17 cells strongly depends on IL-6 [8]. In line with its pleiotropic activities, abrogation of IL-6 signaling has substantial effects on the immune system. Diminished IL-6 signaling causes defects in the control of viral, bacterial and parasite infections [9–11]. Conversely, IL-6 signaling is detrimental in inflammatory diseases such as rheumatoid and juvenile arthritis [12] or proliferative disorders like Castleman’s disease [13].

IL-6 signaling is conveyed through the IL-6 receptor. The receptor consists of the 80 kDa membrane-bound IL-6Rα subunit (CD126), which has no intracellular signaling component, and the signal-transducing gp130 subunit. Two receptors interact with two IL-6 proteins to form the hexameric signaling complex [14]. IL-6’s activities in hematopoiesis, glucose metabolism and in the neuroendocrine system rely on classical IL-6 signaling by this complex [15]. Classical signaling is restricted to cells with expression of the membrane-bound IL-6Rα subunit, including hepatocytes and a subset of leukocytes [16]. IL-6Rα is also found as a soluble protein (sIL-6Rα), which can still bind IL-6. These IL-6/sIL-6Rα complexes are able to associate with gp130 subunits on the cell surface and induce IL-6 signaling. Since gp130 is ubiquitously expressed, this IL-6 trans-signaling substantially broadens the range of IL-6 target cells to all cells in the body [17]. In addition to its membrane-bound form, gp130 is constitutively produced as soluble protein which neutralizes IL-6/sIL-6Rα complexes [18]. Therefore, IL-6 trans-signaling only occurs in situations with secretion of IL-6 and extensive shedding of IL-6Rα. Since sgp130 does not interfere with classical IL-6 signaling, IL-6Rα^+^ cells can still respond to high IL-6 concentrations in the absence of IL-6 trans-signaling. Thus, depending on the concentrations of sIL-6Rα, IL-6 will have distinct effects on the immune system [1]. Recently, a third mode of IL-6 signaling has been described as mechanism to induce T_H_17 cells in the model of experimental autoimmune encephalomyelitis [19]. In dendritic cells (DC), IL-6 can intracellularly bind to the IL-6Rα resulting in IL-6/IL-6Rα complexes on the DC surface. During DC-mediated T-cell interaction, these complexes interact with gp130 on the T-cell surface and induce IL-6 signaling. As a consequence, this IL-6 cluster signaling does not require IL-6Rα on the T cell [19].

The Gram-positive bacterium *Listeria monocytogenes* is a human pathogen that causes listeriosis. Risk groups are individuals undergoing immune suppressive treatment and pregnant women where listeria can cause fatal infection of the fetus [20]. Listeria infection is initially controlled by the innate immune system. Rapid mobilization of myeloid cells from the bone marrow and recruitment of these cells to the sites of infection is essential for the restriction of bacterial replication. Due to its intracytosolic habitat, listeria induce strong T_H_1 and CD8^+^ T-cell responses, and both T-cell subsets are required for pathogen eradication and provide effective protection to re-infection [21].

We could previously show that classical IL-6 signaling is essential for the early control of *L*. *monocytogenes* infection [10], but the target cells and protective mechanisms controlled by IL-6 remained unclear. In the current study, we used mice with IL-6Rα-deficiency restricted to either T cells or myeloid cells to define the role of these cells in IL-6 mediated protection. Abrogation of classical IL-6 signaling in T cells did not interfere with bacteria control or with the induction of specific T_H_1 and CD8^+^ T-cell responses. We could, however, detect a defect in T_H_17-cell differentiation. In contrast, abrogation of classical IL-6 signaling in myeloid cells caused a significant defect in the control of *L*. *monocytogenes*. This defect was not associated with reduced accumulation of granulocytes and inflammatory monocytes at sites of infection, and inflammatory monocytes showed an activated phenotype and were not impaired in the production of inflammatory cytokines. However, inflammatory monocytes showed diminished CD38 expression, a protein associated with the microbicidal M1 macrophage phenotype [22], and a significant reduction in their phagocytic activity, which in turn might be the cause for the impaired control of the infection.

## Material and methods

### Animals

*Il6r*a^fl/fl^ mice (B6.SJL-Il6ra^*tm1*.*1Drew*^/J) [23], CD4^cre^ mice (B6.Cg-Tg[Cd4-cre]1Cwi/BfluJ), LysM^cre^ mice (B6.129P2-*Lyz2*^*tm1(cre)Ifo*^/J), *Il6*KO mice (B6.129S2-*Il6*^*tm1Kopf*^/J) [5], sgp130FcTg mice [24] and wildtype C57BL/6J mice were bred in our facilities. All animals used in this study were on a C57BL/6 background. For all experiments, age and sex matched control mice from the same colony were used. Mouse experiments were carried out in strict accordance with the state guidelines. The protocol was approved by the local ethics committee of the Behörde für Gesundheit und Verbraucherschutz of the City of Hamburg (Permit Number: 81/14). Mice were housed under specific pathogen free conditions in individually ventilated cages with standard food and water ad libitum. During infection experiments, mice were controlled daily and mice with signs of severe disease were euthanized with an O2/CO2 mixture to minimize suffering.

### Animal experiments

Mice were infected with wildtype *Listeria monocytogenes* strain EGD unless stated otherwise. Mice received 2×10^4^ bacteria in 200 μl sterile PBS via the lateral tail vein. Mice were analyzed on day 2 or 3 post-infection (p.i.). For the analysis of primary T-cell responses, mice were i.v. infected with 1×10^4^ ovalbumin-recombinant *L*. *monocytogenes* (LmOVA). T-cell responses were characterized on d8 p.i. For the determination of acquired protection, mice were infected with 2×10^3^ Lm and 7 weeks later, reinfected with 1×10^5^ Lm. Bacterial titers were measured two days later. Bacterial inocula were controlled by serial dilution and plating onto tryptic soy broth (TSB) agar plates. Plates were incubated at 37 °C and colony forming units (CFU) were counted the next day. For *in vivo* phagocytosis analysis, mice were injected with yellow-green fluorescent latex beads diluted 1:25 in PBS (FluoSpheres^®^ Carboxylate-Modified Microspheres, 0.5 μm, Thermo Fisher, Waltham, MA).

For determination of bacterial titers, organs of infected mice were mechanically homogenized in 1 ml 0.1% Triton X-100 in H_2_0 and suspensions were serially diluted. Dilutions were plated on TSB-agar plates and incubated at 37 °C. CFU were counted the next day and bacterial titers in organs were calculated.

### Cytokine profile

Organs of naive and infected mice were collected in RIPA Buffer (150 mM NaCl, 1% NP40, 0,1% Triton X-100, 0,1% SDS, 50 mM Tris-HCl, 5 mM EDTA, [pH 8]) supplemented with cOmplete Mini Protease Inhibitor Cocktail (Roche, Rotkreuz, Switzerland). Organs were mechanically homogenized with the gentleMACS dissociator (Miltenyi Biotec, Bergisch Gladbach, Germany). Cytokines were determined using the LegendPLEX mouse inflammation panel (BioLegend, San Diego, CA) according to manufacturer’s instruction.

Whole RNA was obtained by homogenizing tissue samples and extracting RNA using the Nucleospin^®^ RNA Kit (Macherey-Nagel, Düren, Germany). cDNA was transcribed using the high-capacity cDNA reverse transcription kit (Thermo Fisher). Quantitative PCR was performed with the SYBR^®^ Green JumpStart^™^ Taq ReadyMix^™^ (Sigma-Aldrich) on a StepOnePlus^™^ real-time PCR system (Thermo fisher). Results were normalized to 18S RNA using the ΔCT method. qPCR primers: *Il1b* forward: AAC CTG CTG GTG TGT GAC GTT C, *Il1b* reverse: CAG CAC GAG GCT TTT TTG TTG T, *Il6* forward: TGG GAA ATC GTG GAA ATG AGA, *Il6* reverse: AAG TGC ATC ATC GTT GTT CATA CA, *Nos2* forward: GCT GTT CTC AGC CCA ACA AT, *Nos2* reverse: TTC TGT GCT GTC CCA GTG AG, *18S* forward: CAC GGC CGG TAC AGT GAA AC, 18S reverse: AGA GGA GCG AGC GAC CAA A.

### Cell isolation

Spleens were mashed through a cell sieve to prepare a single cell suspension. Erythrocytes were lysed by incubation in ammonium chloride potassium (ACK) lysis buffer (155 mM NH_4_Cl, 10 mM KHCO_3_, 100 μM EDTA [pH 7.2]). Liver cells were additionally purified using a one-step Easycoll gradient (Biochrom AG, Berlin, Germany) before erythrocyte lysis. Cells were counted with an automated cell counter.

### *In vitro* stimulation of primary murine cells

2×10^6^ cells were incubated in 1ml of standard medium (Iscove’s modified Dulbecco’s medium (IMDM), 5% fetal calf serum, 50 μg/ml gentamicin, 50 μM 2-mercaptoethanol, 200 μM L-glutamine) containing stimulants. T cells were stimulated polyclonally with phorbol-12-myristate-13-acetate (PMA, 50 ng/ml) and ionomycin (1 μM), or antigen-specific with listeriolysin O peptide 189–201 (LLO, 10^−5^ M, JPT, Berlin, Germany) and ovalbumin peptide 257–264 (OVA, 10^−6^ M, JPT). For stimulation of monocytes, lipopolysaccharide from *E*. *Coli* 055:B5 (LPS, 1 μg/ml, Sigma-Aldrich, St. Louis, MO) was utilized. Cells were incubated for 30 minutes at 37 °C and 5% CO_2_. Brefeldin A (10 μg/ml) was added to block the Golgi apparatus. After brefeldin A addition, the cells were incubated for additional 210 minutes.

### Generation of bone marrow-derived macrophages (BMDM)

Bone marrow was isolated from femur and tibia of naive mice. Cells were counted and resuspended in BMDM medium (Dulbecco’s modified Eagle medium (DMEM), 10% fetal calf serum, 5% horse serum, 10 ng/μl M-CSF (Peprotech, Rocky Hill, NJ), 1 mM HEPES, 100 μM sodium pyruvate, 50 μg/ml gentamicin, 200 μM L-glutamine) at a concentration of 5×10^5^ cells/ml. 10 ml of cell suspension was cultured in a petri dish for 5 days at 37 °C and 5% CO_2_. Cells were supplemented with 5 ml BMDM medium and incubated for 3 additional days. Cells were mechanically detached from the petri dishes and resuspended in standard medium at a concentration of 5×10^5^ cells/ml. 1 ml of cell suspension per well was seeded onto a 24-well plate. For M1 and M2 polarization, cells were supplemented with either LPS (2 μg/ml, Sigma-Aldrich) and recombinant IFNγ (40 ng/ml) or recombinant IL-4 (40 ng/ml). Cells were additionally incubated with or without recombinant IL-6 (160 ng/ml, Stefan Rose-John, CAU, Kiel) for 24 h. To induce NO production, cells were further stimulated with heat-killed listeria (5×10^6^) for the final 18 h. Supernatant was collected and cells were detached by incubation in PBS with 0.25 mM EDTA and kept on ice until staining. Listeria uptake and control of intracellular replication in BMDM was assessed as described before [25]. NO concentrations were determined using a commercially available kit (Promega, Fitchburg, WI) according to the manufacturer’s instruction. 50 μl undiluted culture supernatant was used for analysis.

### Immuno-fluorescent staining for flow cytometry

Non-specific antibody binding was blocked by incubation of cells with rat serum and Fc receptor blocking antibodies (clone 2.4G2, Bio X Cell, West Lebanon, NH). Dead cells were excluded by staining with pacific orange succinimidyl ester (Thermo Fischer). Cells were extracellularly stained with antibodies (S1 Table). For intracellular cytokine staining, cells were fixed in 2% paraformaldehyde and non-specific staining was blocked with rat serum diluted in saponin buffer (PBS, 0.1% BSA, 0.45 g saponin (Sigma-Aldrich)). Intracellular cytokine staining was performed using antibodies diluted in saponin buffer. Stained cells were analyzed with a BD FACS CANTO II (Becton Dickinson) using BD FACS DIVA 8.0 (Becton Dickinson) and Flowjo vX (Flowjo, Ashland, OR). The gating strategy is given in S1A and S1B Fig.

### Chemotaxis assay

Chemotaxis index was determined by a transwell assay using 24-well transwell plates with a 5 μm pore size (Costar, Corning, NY). 1×10^6^ spleen cells from infected mice were loaded in standard medium into the top chamber. CCL2 (0.15 ng/μl) or fMLP (N-formyl-met-leu-phe, 12.5 μM) were used as chemotactic agents in the lower chamber. Cells were incubated for 2 h at 37 °C and 5% CO_2_. Migrated cells were analyzed by flow cytometry.

### Immunohistochemistry

Tissue was fixed overnight in 4% buffered formalin and embedded in paraffin. Macrophages and inflammatory monocytes were stained with an antibody specific for F4/80 (BM8, BioLegend) developed with the DAKO EnVision^™^+ system (Agilent, Santa Clara, CA) and counterstained with Mayer‘s Hematoxylin Solution. 5–15 pictures were taken per slide at 10x magnification. F4/80-positive area was calculated with ImageJ (NIH, Bethesda, MD).

### Statistical analysis

Statistical analysis was performed with Prism Software (GraphPad Software, La Jolla, CA). In titers experiments, median values are represented by a line and differences were calculated by Mann-Whitney U test. In all other experiments, bars or lines represent means and error bars represent SEM. Differences were calculated either with student‘s t-test or 2way ANOVA with Bonferroni’s post-test. A P value of <0.05 was considered significant (* P<0.05; ** P<0.01; *** P<0.001).

## Results

### Classical IL-6 signaling in T cells is not required for the control of *Listeria monocytogenes*

**Fig 1:**
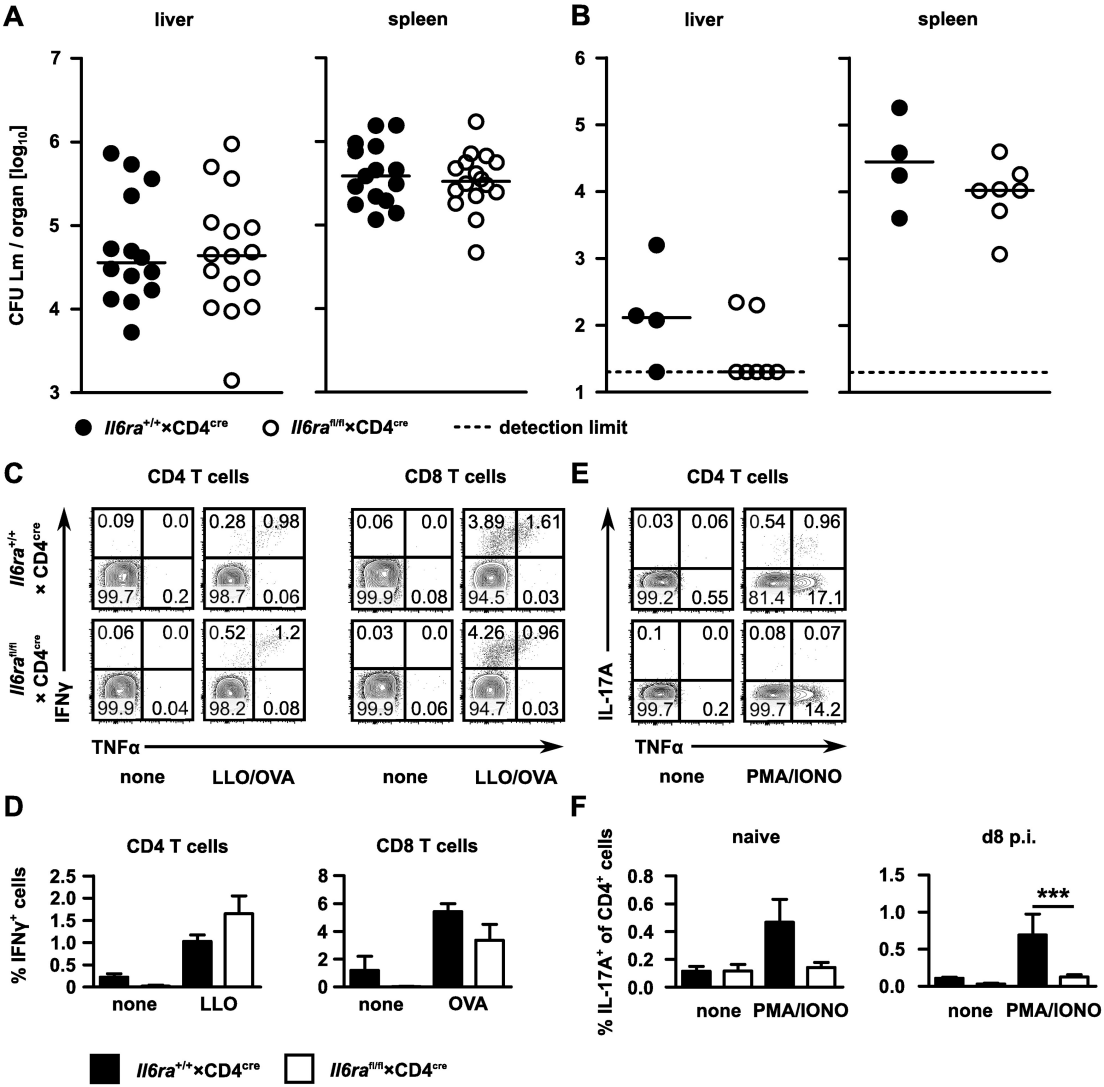
Classical IL-6 signaling in T cells has only limited impact on T-cell differentiation and bacteria control during *Listeria monocytogenes* infection. *Il6ra*^fl/fl^×CD4^cre^ and CD4^cre^ control mice were infected i.v. with 2×10^4^ listeria. Two days p.i., titers in liver and spleen were determined (**A**). Results for individually analyzed mice and median bars are shown. Significance was determined with the Mann-Whitney U test. Results from two independent experiments were pooled. Mice were i.v. infected with 2×10^3^ and after 7 weeks with 1×10^5^ listeria. Titers were determined two days post reinfection (**B**). Significance was determined with the Mann-Whitney U test. One representative experiment of two is shown. *Il6ra*^fl/fl^×CD4^cre^ and control mice were infected i.v. with 1×10^4^ listeria recombinant for ovalbumin. Eight days p.i., spleen cells were isolated and stimulated with LLO_189-201_ and OVA_257-264_ (**C, D**), or ionomycin and PMA (**E, F**). Intracellular IFNγ, TNFα and IL-17A expression was determined by flow cytometry. (**C**) Representative dot plots for CD4^+^ and CD8^+^ spleen cells from infected mice, with or without peptide stimulation. (**D**) Frequencies of IFNγ^+^CD4^+^ or IFNγ^+^CD8^+^ cells from spleens of infected *Il6ra*^fl/fl^×CD4^cre^ and control mice following *in vitro* stimulation. (**E**) Representative dot plots for CD4^+^ spleen cells from infected mice, with or without polyclonal stimulation. (**F**) Frequencies of IL-17A^+^CD4^+^ cells from spleens of naive and infected *Il6ra*^fl/fl^×CD4^cre^ and control mice following *in vitro* stimulation. Bars present mean ± SEM of 4 mice per group. Significance was determined with student’s t-test. One representative experiment of two is shown.

The cytokine IL-6 has proinflammatory activities and is required for the control of bacterial infection [6, 26]. In a previous study, we could demonstrate that the control of *Listeria monocytogenes* depends on classical IL-6 signaling but not on IL-6 trans-signaling [10]. To characterize the mechanisms of IL-6-mediated protection and to identify the responsible IL-6 target cells, we used mice with a deficiency of IL-6Rα in CD4^+^ and CD8^+^ T cells (*Il6ra*^fl/fl^×CD4^cre^ mice) or in myeloid cells (*Il6ra*^fl/fl^×LysM^cre^ mice). In a first set of experiments, we used *Il6ra*^fl/fl^×CD4^cre^ mice to test the role of classical IL-6 signaling in T cells in the control of *L*. *monocytogenes*. *Il6ra*^fl/fl^×CD4^cre^ mice showed effective reduction of IL-6Rα surface expression on CD4^+^ and CD8^+^ T cells (S1C Fig). *Il6ra*^fl/fl^×CD4^cre^ and control mice (CD4^cre^) were infected i.v. with 2×10^4^ listeria. Two days p.i., the mice were sacrificed and the bacterial titers in spleen and liver were determined. Abrogation of classic IL-6 signaling in T cells showed no effect on the bacterial burden in spleen and liver (Fig 1A).

Next, we analyzed the influence of classical IL-6 signaling in T cells on the specific T-cell responses against *L*. *monocytogenes*. *Il6ra*^fl/fl^×CD4^cre^ and control mice were infected i.v. with 2×10^4^ ovalbumin-recombinant listeria (LmOVA) and sacrificed 8 days p.i. Spleen cells were stimulated with the peptides listeriolysin O_189-201_ and ovalbumin_257-264_ which are immunodominant for CD4^+^ and CD8^+^ T cells, respectively. Frequencies of IFNγ^+^ and TNFα^+^ CD4^+^ and CD8^+^ T cells were determined by flow cytometry. Impaired classical IL-6 signaling had no effect on the frequencies of antigen-specific IFNγ or TNFα producing CD4^+^ or CD8^+^ T cells (Fig 1C and 1D, and not shown). Consistent with this result, we did not detect significant changes in the specific T-cell response to *L*. *monocytogenes* in *Il6*KO mice and in sgp130FcTg mice, transgenic for a soluble gp130 receptor which neutralizes soluble IL-6Rα/IL-6 complexes and thereby inhibits IL-6 trans-signaling [24] (S2A and S2B Fig).

Control of secondary *L*. *monocytogenes* infection depends on T cells. *Il6ra*^fl/fl^×CD4^cre^ and control mice were infected and after 7 weeks, mice were reinfected with a high dose of *L*. *monocytogenes*. Determination of listeria titers in spleen and liver 2 days post reinfection revealed similar control of the secondary infection in both mouse strains (Fig 1B). Thus, classical IL-6 signaling was not required for protective T-cell responses against *L*. *monocytogenes*.

### Classical IL-6 signaling is responsible for T_H_17-cell differentiation during infection

T_H_17-cell differentiation is dependent on IL-6, but the role of classical IL-6 signaling is still controversial. *Il6ra*^fl/fl^×CD4^cre^ and control mice were infected i.v. with 2×10^4^ LmOVA and sacrificed 8 days p.i. Upon polyclonal stimulation with PMA and ionomycin, we observed significantly reduced frequencies of IL-17A^+^ CD4^+^ T cells in naive and infected *Il6ra*^fl/fl^×CD4^cre^ mice when compared to control mice (Fig 1E and 1F). The same effect was seen after stimulation of spleen cells from *Il6*KO mice but not from sgp130FcTg mice (S2C and S2D Fig). Taken together, these results show that classical but not IL-6 trans-signaling is responsible for T_H_17-cell differentiation during *L*. *monocytogenes* infection.

### Control of *Listeria monocytogenes* depends on classical IL-6 signaling in myeloid cells

Since IL-6 signaling had no significant effect on the adaptive immune response against *L*. *monocytogenes*, we decided to focus on the innate immune response using *Il6ra*^fl/fl^×LysM^cre^ mice. Inflammatory monocytes showed surface expression of IL-6Rα which was substantially reduced in *Il6ra*^fl/fl^×LysM^cre^ mice (S1C Fig). In contrast, granulocytes, macrophages and non-classical Ly6C^low^ monocytes expressed only relatively low levels of surface IL-6Rα and the expression was not further diminished in *Il6ra*^fl/fl^×LysM^cre^ mice (S1C Fig).

**Fig 2:**
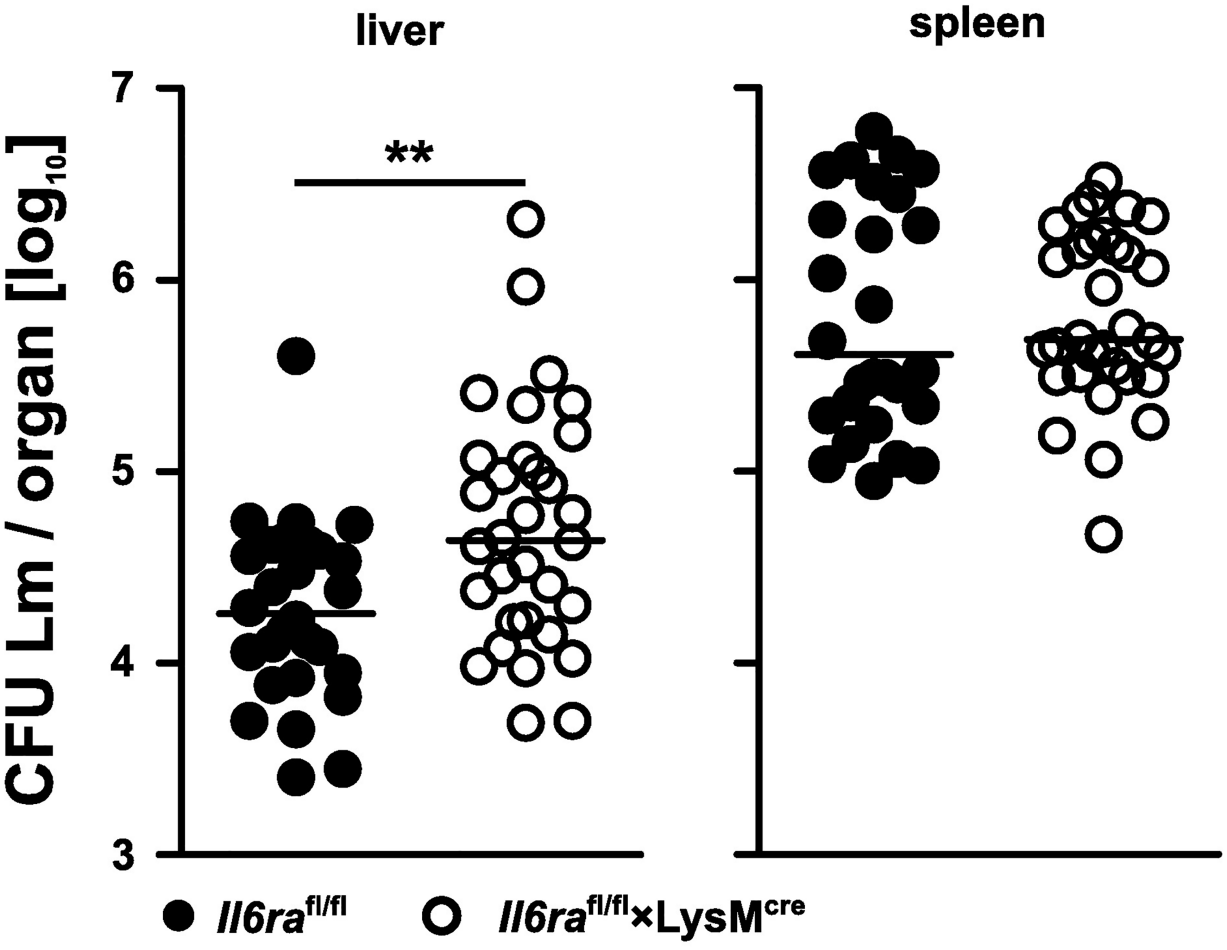
Control of *Listeria monocytogenes* depends on classical IL-6 signaling in myeloid cells. *Il6ra*^fl/fl^×LysM^cre^ mice and *Il6ra*^fl/fl^ littermate controls were infected i.v. with 2×10^4^ listeria. Two days p.i., titers in livers and spleens were determined. Data was pooled from 4 independent experiments. Results for individually analyzed mice and median bars are shown Significance was determined with the Mann-Whitney U test.

*Il6ra*^fl/fl^×LysM^cre^ and littermate control mice (*Il6ra*^fl/fl^) were infected with 2×10^4^ listeria. Two days p.i., bacterial titers in livers and spleens were determined. Titers were significantly increased in livers of *Il6ra*^fl/fl^×LysM^cre^ mice. Titers in spleens of *Il6ra*^fl/fl^×LysM^cre^ mice were similar to those of control mice (Fig 2). Higher titers were still detected *Il6ra*^fl/fl^×LysM^cre^ mice at day 5 p.i. (S3A Fig).

*Il6ra*^fl/fl^×LysM^cre^ and control mice were also infected with LmOVA and eight days later, frequencies of T cells responding to the peptides listeriolysin O_189-201_ and ovalbumin_257-264_ were determined (S3B Fig). Spleens of both groups of mice showed similar frequencies for IFNγ^+^ CD8^+^ T cells. However, we observed significantly higher frequencies of IFNγ^+^ CD4^+^ T cells in *Il6ra*^fl/fl^×LysM^cre^ mice. Thus, absence of classical IL-6 signaling in myeloid cells did not impair but rather enhance the T-cell response to infection.

### Effect of myeloid cell-specific IL-6Rα abrogation on the innate immune response

*Il6ra*^fl/fl^×LysM^cre^ mice and littermate controls were infected i.v. with 2×10^4^ listeria. Mice were sacrificed 2 days p.i. and concentrations of inflammatory cytokines were determined in tissue extracts from spleen and liver (S4A–S4D Fig). In infected mice, we observed an increase in TNFα, IFNγ, IL-1α, GM-CSF, CCL2, IL-6 and IL-1β which was generally more pronounced in spleen compared to liver. However, changes were comparable in *Il6ra*^fl/fl^×LysM^cre^ and control mice. Other cytokines (IL-12p70, IL-10, IL-17A) did not or only marginally change following infection or were even reduced (IL-23, IFNβ). Interestingly, some cytokines such as IL-12p70, IL-23, IL-17A and IFNβ, were slightly higher expressed in naive and/or infected *Il6ra*^fl/fl^×LysM^cre^ mice compared to control mice. In summary, infected *Il6ra*^fl/fl^×LysM^cre^ and control mice showed only minor differences in their cytokine profile.

Hepatic expression of IL-1β, IL-6 and iNOS was assessed on mRNA level (S4E–S4G Fig). *Il1b*, *Nos2* (coding for iNOS) and *Il6* were upregulated in infection, however, there was similar upregulation in both mouse strains.

**Fig 3:**
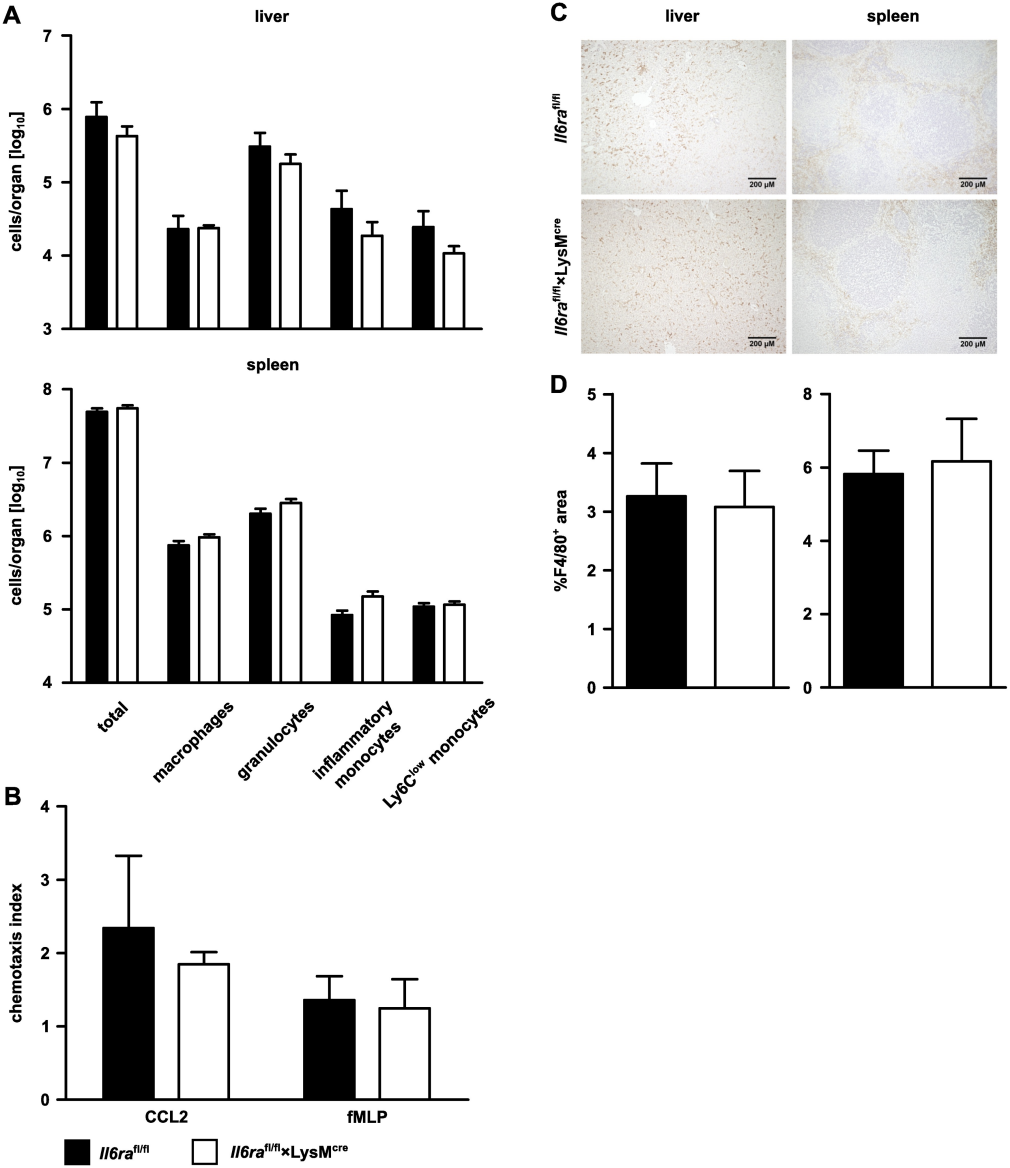
Distribution of inflammatory monocytes during the early phase of listeria infection is unaffected by abrogated classical IL-6 signaling. *Il6ra*^fl/fl^×LysM^cre^ and *Il6ra*^fl/fl^ littermate control mice were infected i.v. with 2×10^4^ listeria. Mice were analyzed 2 (**A** and **B**) or 3 days (**C** and **D**) p.i. (**A**) Liver and spleen cells were isolated and total leukocyte numbers as well as numbers of macrophages, granulocytes, inflammatory monocytes and Ly6C^low^ monocytes were determined. Groups contained 6 to 7 mice. Representative results of one of two experiments are shown. (**B**) Chemotaxis index for inflammatory monocytes was determined by transwell assay. Migration was induced by CCL2 and fMLP. One representative experiment of two with two replicates per group is shown. (**C**) F4/80 staining of paraffin fixed liver and spleen sections. Representative pictures of liver and spleen from infected mice are shown. (**D**) Statistical analysis of F4/80-positive areas from liver and spleen sections of infected mice. Groups contained 5 to 7 mice. Bars represent mean ± SEM. Significance was determined by unpaired student’s t-test.

Following *L*. *monocytogenes* infection, granulocytes and inflammatory monocytes rapidly accumulate in the infected tissues and particularly inflammatory monocytes are crucial for the control of infection [27, 28]. To test whether abrogated IL-6 signaling in *Il6ra*^fl/fl^×LysM^cre^ mice resulted in an impaired response of inflammatory monocytes or granulocytes, *Il6ra*^fl/fl^×LysM^cre^ mice and littermate controls were infected i.v. with 2×10^4^ listeria. Two days p.i., the distribution of various myeloid cell populations in spleen and liver was determined by flow cytometry. We did not detect differences in the total leukocyte numbers as well as in the numbers of granulocytes, inflammatory monocytes, Ly6c^low^ monocytes and macrophages between infected *Il6ra*^fl/fl^×LysM^cre^ and control mice (Fig 3A, Gating strategy in S1A Fig). Since inflammatory monocytes showed the most pronounced shift in IL-6Rα expression, we focused in subsequent analyses mainly on these cells. In order to study the ability of the inflammatory monocytes to move into the infected organs, the migratory capacity of these cells was directly tested in a chemotaxis assay. Total spleen cells from infected mice were subjected to gradients of CCL2 or fMLP, and the migration of inflammatory monocytes towards high CCL2 and fMLP concentration was determined. The chemokine CCL2 is a ligand of the chemokine receptor CCR2, which plays a major role in mobilization of inflammatory monocytes in response to infection [28]. fMLP is a ligand of the formyl peptide receptor 1 (FPR1), which recognizes formyl methionine-containing bacterial peptides [29, 30]. For both chemotactic factors, we observed no difference in the chemotactic index between inflammatory monocytes from infected *Il6ra*^fl/fl^×LysM^cre^ and control mice (Fig 3B). Finally, livers and spleens from infected mice were analyzed for the accumulation of F4/80^+^ cells by immunohistochemistry. We did not observe a significant difference in the F4/80-positive area between *Il6ra*^fl/fl^×LysM^cre^ mice and littermate controls (Fig 3C and 3D).

### IL-6 shapes the differentiation of inflammatory monocytes

**Fig 4:**
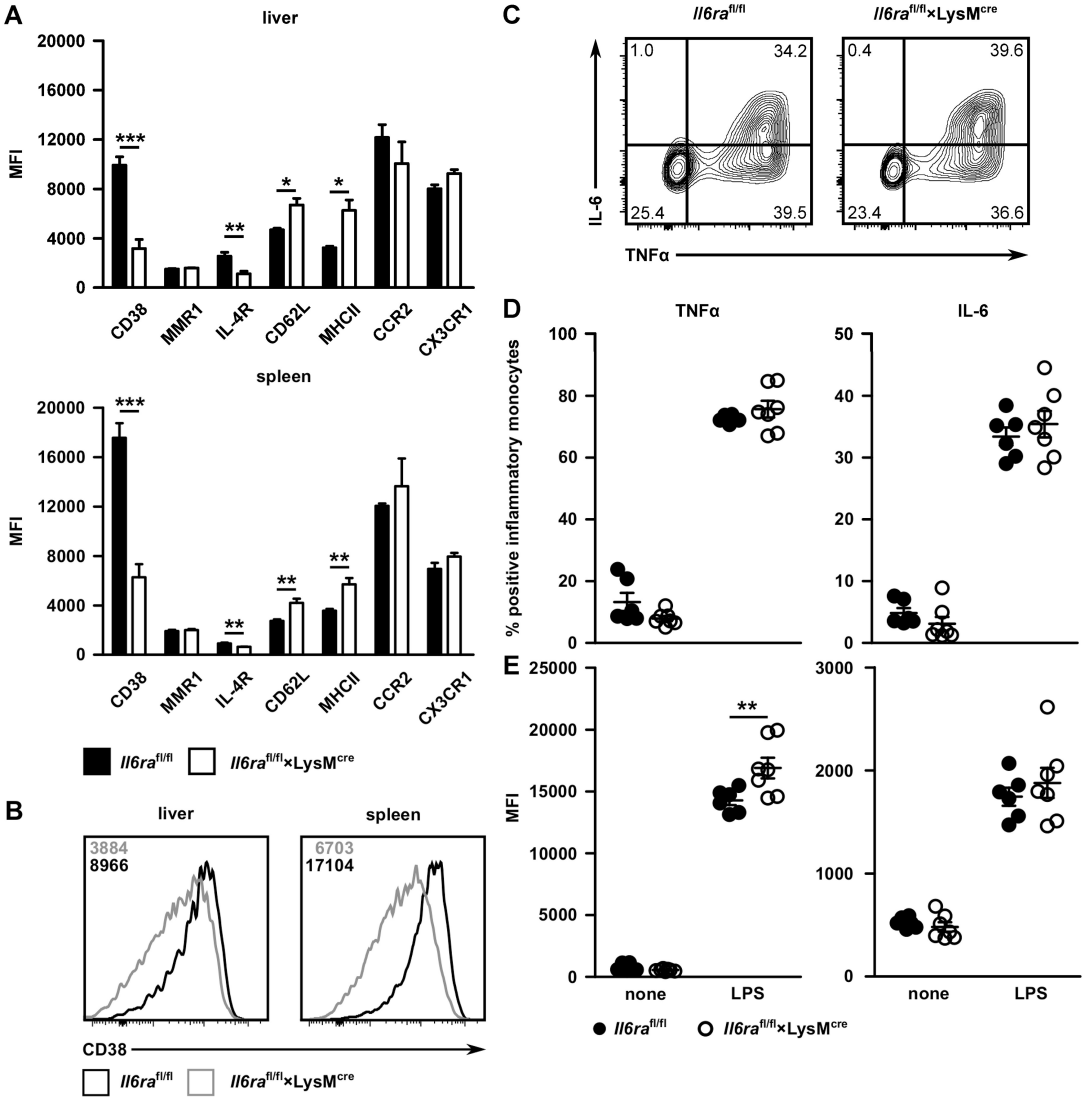
IL-6 affects differentiation of inflammatory monocytes. *Il6ra*^fl/fl^×LysM^cre^ and *Il6ra*^fl/fl^ littermate control mice were infected i.v. with 2×10^4^ listeria. Two days p.i., liver and spleen cells from infected mice were isolated and inflammatory monocytes were characterized by flow cytometry (gating strategy is given in S1A Fig). (**A**) Mean fluorescence intensity (MFI) of different surface proteins. (**B**) Representative histograms of CD38 expression. (**C**) Representative dot plots of intracellular TNFα and IL-6 expression in inflammatory monocytes. Spleen cells were stimulated with LPS for 4 h. (**D**) Frequency of TNFα^+^ and IL-6^+^ inflammatory monocytes. (**E**) MFI of intracellular TNFα and IL-6 staining of inflammatory monocytes. One representative experiment of two with 5 to 7 mice per group is shown. Bars represent mean ± SEM. Significance was determined with student’s t-test.

IL-6 is involved in differentiation and polarization of monocytes and macrophages [4]. Thus, it is possible that deficiency of classical IL-6 signaling in myeloid cells causes altered maturation of inflammatory monocytes during *L*. *monocytogenes* infection. *Il6ra*^fl/fl^×LysM^cre^ mice and littermate controls were infected i.v. with 2×10^4^ listeria. Two days p.i., inflammatory monocytes from spleen and liver were analyzed for the expression of surface proteins associated with activation, differentiation and function (Fig 4A). Flow cytometric analysis revealed higher abundance of CD62L and MHC II on inflammatory monocytes from *Il6ra*^fl/fl^×LysM^cre^ mice, suggesting a more activated status of these cells [31]. In contrast, the chemokine receptors CCR2 and CX3CR1 were similarly expressed on inflammatory monocytes from infected *Il6ra*^fl/fl^×LysM^cre^ and control mice. Macrophage mannose receptor 1 (MMR1) and IL-4Rα are upregulated on M2 and CD38 on M1 macrophages [4, 22]. Since IL-6 was described to promote the maturation to M2 macrophages, we analyzed the expression of these proteins on inflammatory monocytes. We observed similar expression of the MMR1 on inflammatory monocytes from infected *Il6ra*^fl/fl^×LysM^cre^ and control mice. In contrast, surface expression of IL-4Rα and particularly of CD38 was reduced on inflammatory monocytes from *Il6ra*^fl/fl^×LysM^cre^ mice (Fig 4A and 4B).

Spleen cells were also stimulated with LPS and the production of TNFα and IL-6 by inflammatory monocytes was determined by intracellular cytokine staining. TNFα and IL-6 were produced by similar frequencies of inflammatory monocytes of both mouse strains. However, we observed a higher staining intensity in inflammatory monocytes from *Il6ra*^fl/fl^×LysM^cre^ mice indicating that individual cells produced more TNFα (Fig 4C–4E).

Overall, inflammatory monocytes from infected *Il6ra*^fl/fl^×LysM^cre^ mice displayed higher levels of the activation markers MHC II and CD62L and produced more TNFα upon activation. On the other hand, IL-6Rα lacking cells showed reduced surface expression of IL-4Rα but also of CD38, suggesting that absence of classical IL-6 signaling altered their maturation.

Expression of surface markers and cytokines was also assessed on granulocytes (S5A–S5D Fig) and Ly6C^low^ monocytes (S5E–S5H Fig). Compared to inflammatory monocytes, granulocytes displayed lower expression of all analyzed surface markers. We observed small changes in the expression of CD38, IL-4Rα, CD62L and CX3CR1 in granulocytes from *Il6ra*^fl/fl^×LysM^cre^ mice. Ly6C^low^ monocytes from *Il6ra*^fl/fl^×LysM^cre^ mice had diminished CD38 expression. All other markers did not differ between cells from both mouse lines. Compared to inflammatory monocytes, granulocytes and Ly6C^low^ monocytes produced less TNFα and IL-6, in terms of both frequencies of positive cells and staining intensity for cytokines. We detected similar expression of TNFα in cells from both mouse strains, but fewer Ly6C^low^ monocytes produced IL-6.

### IL-6 regulates maturation of bone marrow-derived macrophages

**Fig 5:**
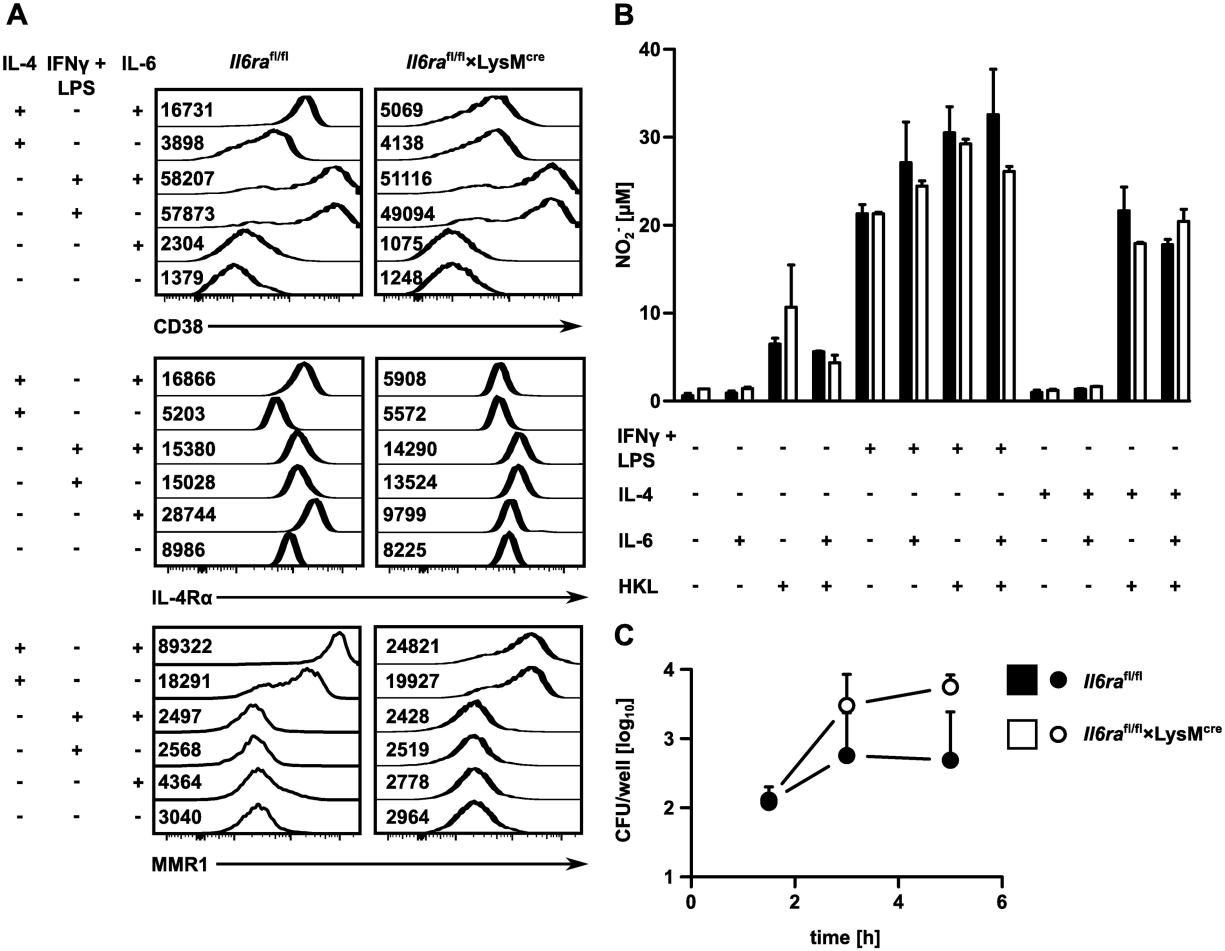
IL-6 regulates maturation of bone marrow-derived macrophages. (**A**) BMDM from *Il6ra*^fl/fl^×LysM^cre^ and *Il6ra*^fl/fl^ control mice were differentiated with IL-4 or LPS + IFNγ and stimulated overnight with IL-6. Surface expression of CD38, IL-4Rα and MMR1 was analyzed by flow cytometry. Histograms and MFI-values are shown. (**B**) BMDM were stimulated as described before. Heat-killed listeria were added for the final 18 hours. NO_2_^-^ concentration in supernatants was determined in duplicates. (**C**) BMDM were infected *in vitro* with listeria (Strain EGD, MOI 10 1), extracellular bacteria were removed with gentamicin and titers were determined at indicated time points. One representative experiment of two is shown. Bars in (**B**) represent mean ± SEM and circles in (**C**) median ± range. Significance was determined with (**B**) student’s t-test or (**C**) Mann-Whitney U test.

To directly determine the impact of IL-6 on the expression on MMR1, IL-4Rα and CD38, BMDM were cultured under M0 (no additional stimulus), M1 (IFNγ + LPS) or M2 (IL-4) polarizing conditions and were subsequently incubated overnight with or without IL-6 (Fig 5A). We observed only low surface expression of CD38 under M0 conditions and expression was slightly increased by IL-6 in macrophages from control mice but not from *Il6ra*^fl/fl^×LysM^cre^ mice. CD38 was strongly induced under M1 conditions and this induction was independent from IL-6 or the expression of the IL-6Rα. CD38 was also induced under M2 conditions and here, induction required IL-6 and IL-6Rα. Induction of IL-4Rα was observed in cells from control mice under M0 and M2 conditions. Under M1 conditions, macrophages showed slightly enhanced expression, which was independent from IL-6. MMR1 was induced only under M2 conditions and expression was further increased in cells from control mice when treated with IL-6.

BMDM from *Il6ra*^fl/fl^×LysM^cre^ and control mice were also used to test the role of IL-6 in NO production. M0, M1 and M2 macrophages were stimulated with IL-6 and heat-killed listeria (HKL), and NO in supernatant was determined (Fig 5B). We observed strong NO production by M1 macrophages, which was only marginally increased by HKL. In contrast, M0 and M2 macrophages secreted NO only after HKL stimulation. However, under all conditions NO production was independent from both the expression of the IL-6Rα and the stimulation with IL-6.

### Phagocytic potential of inflammatory monocytes is diminished in mice with abrogated classical IL-6 signaling in myeloid cells

Phagocytosis of bacteria is a central mechanism of myeloid cells for the control of bacterial infection. Therefore, we decided to analyze phagocytosis and killing of bacteria by IL-6Rα-deficient myeloid cells. BMDM from *Il6ra*^fl/fl^×LysM^cre^ and *Il6ra*^fl/fl^ control mice were infected with listeria. Extracellular listeria were killed with gentamicin and the numbers of intracellular listeria were determined at different time points of infection (Fig 5C). BMDM from both mouse strains showed similar listeria uptake. However, at later time points we could recover higher numbers of listeria from BMDM from *Il6ra*^fl/fl^×LysM^cre^ mice, indicating impaired control of listeria replication in these cells.

**Fig 6:**
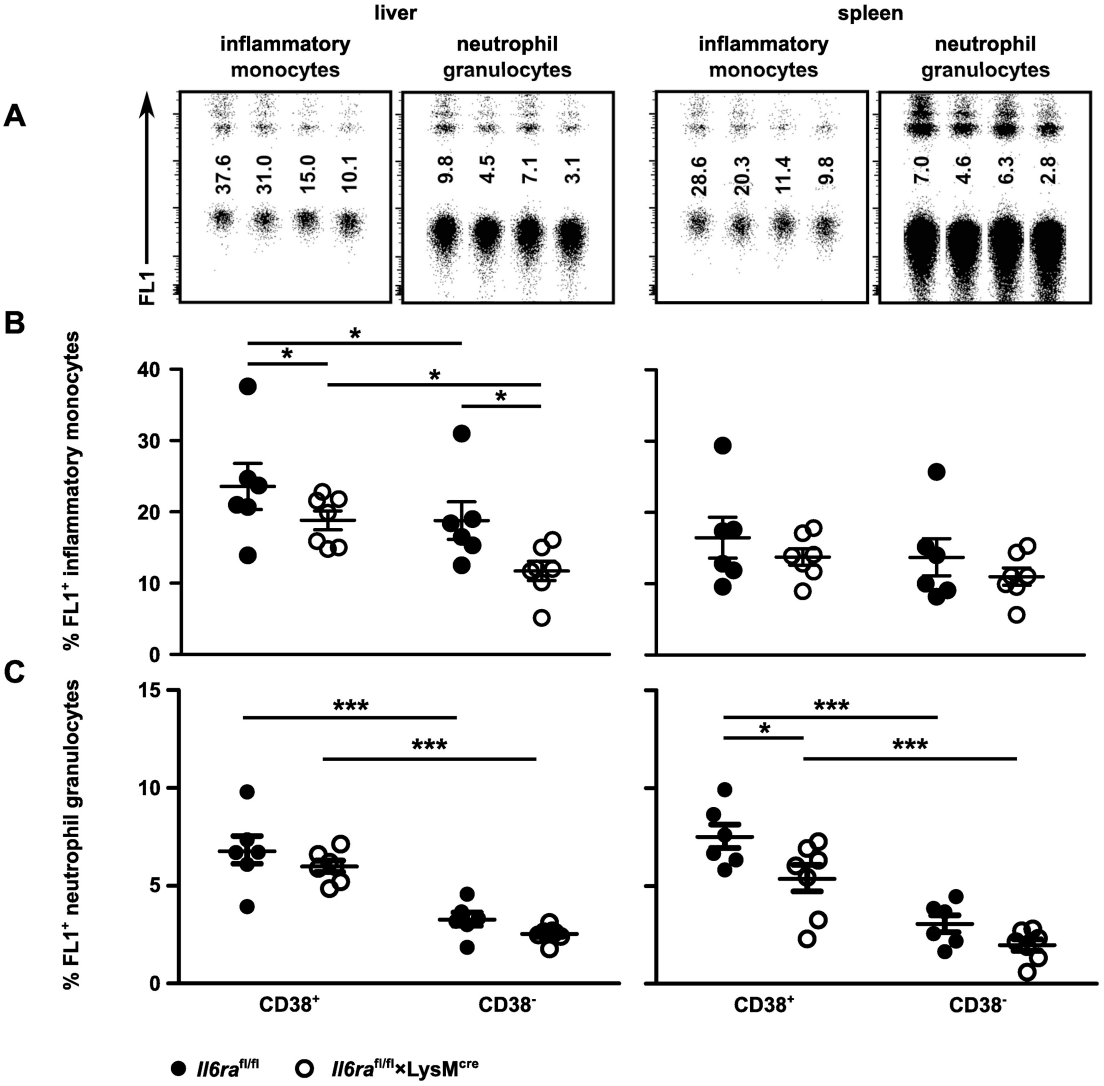
Phagocytic potential of inflammatory monocytes is diminished in mice with abrogated classical IL-6 signaling in myeloid cells. *Il6ra*^fl/fl^×LysM^cre^ and *Il6ra*^fl/fl^ littermate controls were infected i.v. with 2×10^4^ listeria. Two days p.i., mice were injected i.v. with fluorescently labelled latex beads. Liver and spleen cells were isolated 1 hour after injection. Uptake of latex beads by inflammatory monocytes and neutrophil granulocytes was evaluated by flow cytometry. (**A**) Representative dot plots (left to right: *Il6ra*^fl/fl^ CD38^+^, *Il6ra*^fl/fl^ CD38^-^, *Il6ra*^fl/fl^×LysM^cre^ CD38^+^, *Il6ra*^fl/fl^×LysM^cre^ CD38^-^) of inflammatory monocytes or neutrophil granulocytes from liver and spleen of infected mice. Numbers give the %-value of latex bead^+^ cells. (**B**) Frequencies of latex bead^+^ CD38^+^ and CD38^-^ inflammatory monocytes from liver and spleen of infected mice. (**C**) Frequencies of latex bead^+^ CD38^+^ and CD38^-^ neutrophil granulocytes from liver and spleen of infected mice. One representative experiment of three with 4 to 7 mice per group is shown. Mean ± SEM are shown. Significance was determined by 2way ANOVA.

To test the phagocytosis *in vivo*, *Il6ra*^fl/fl^×LysM^cre^ and littermate control mice were infected i.v. with 2×10^4^ listeria. Two days p.i., mice were injected i.v. with fluorescent latex beads. The mice were sacrificed 1 h later, liver and spleen cells were isolated, and uptake of latex beads by inflammatory monocytes and granulocytes was analyzed by flow cytometry (Fig 6). We could recently demonstrate that CD38-deficiency causes enhanced susceptibility of mice to *L*. *monocytogenes* infection, which was largely due to an impaired innate response [25]. Furthermore, it could be shown that abrogation of CD38 signaling can lead to reduced phagocytosis [32]. Therefore, we determined the uptake of beads in CD38^+^ and CD38^-^ cells. In both mouse strains, CD38^+^ inflammatory monocytes were more efficient in the uptake of latex beads. Uptake of beads was significantly reduced in CD38^+^ and CD38^-^ inflammatory monocytes from the liver of *Il6ra*^fl/fl^×LysM^cre^ mice when compared to corresponding cells from littermate controls. Compared to the liver, we detected less bead-positive cells in the spleen. CD38^+^ control cells were slightly more efficient in phagocytosis, however, differences did not reach significance in the spleen. Granulocytes were less efficient in phagocytosis of beads, with very little phagocytosis by CD38^-^ granulocytes. For these cells, we detected lower levels of phagocytosis in the spleen.

In conclusion, abrogation of classical IL-6 signaling had a direct effect on the phagocytic activity, particularly in CD38^+^ inflammatory monocytes. The low expression level of CD38 on inflammatory monocytes from infected *Il6ra*^fl/fl^×LysM^cre^ mice could further enhance the deficiency in phagocytosis.

## Discussion

Infection of mice with *L*. *monocytogenes* causes strong induction of IL-6 which is required for effective bacteria control [6, 10, 26]. We could recently demonstrate that this control depends on classical IL-6 signaling but not on IL-6 trans-signaling [10]. In the current study, we aimed at identifying the target cells of IL-6 responsible for listeria control.

Deletion of IL-6Rα in CD4^+^ and CD8^+^ T cells did not alter the control of *L*. *monocytogenes* since we observed similar bacterial titers in organs of *Il6ra*^fl/fl^×CD4^cre^ and control mice after primary and secondary infections. Studies on the function of IL-6 in the formation of T_H_1 cells and CD8^+^ T cells yielded inconsistent results [9, 33–36]. It could be demonstrated that IL-6 represses T_H_1-cell responses by induction of T_H_2 cells and blockade of IL-12 and IFNγ signaling [37, 38]. However, IL-6-deficient mice fail to mount T_H_1-cell responses to bacterial and fungal infections [35, 39]. Furthermore, IL-6 can prevent T_reg_-cell mediated suppression of T_H_1 cells and thereby even supports T_H_1-cell responses [36, 40]. Absence of IL-6 enhances CD8^+^ T-cell responses against protozoan infection but has either no effect on or even restricts CD8^+^ T-cell responses to viral infections [9, 40, 41]. Finally, there is evidence that IL-6 supports the induction of cytotoxic function in CD8^+^ T cells [42, 43]. In our listeria infection model, *Il6ra*^fl/fl^×CD4^cre^ and control mice showed similar frequencies of CD4^+^ and CD8^+^ T cells responding to stimulation with immunodominant peptides. Although we consistently detect IL-6Rα on naive CD4^+^ and CD8^+^ T cells, surface IL-6Rα expression is rapidly lost from these cells upon *in vitro* stimulation or *in vivo* following *L*. *monocytogenes* infection [10, 44]. Thus, T cells might rely on IL-6 trans-signaling by soluble IL-6Rα/IL-6 complexes or IL-6 cluster-signaling by IL-6Rα^+^ antigen-presenting cells [18, 43, 45]. However, sgp130FcTg mice with impaired IL-6 trans-signaling and *Il6*KO mice with a complete lack of IL-6 signaling generated normal CD4^+^ T_H_1 and CD8^+^ T-cell responses to *L*. *monocytogenes* indicating that IL-6 in general was not required for the effective formation of listeria-specific T cells. The cause for the apparent lack of IL-6 activity in our model is unclear. *L*. *monocytogenes* induces a strong inflammatory response and a wide spectrum of pro-inflammatory cytokines which might compensate for the missing IL-6 signal in T cells. Interestingly, we observed even stronger CD4^+^ T-cell responses in *Il6ra*^fl/fl^×LysM^cre^ mice. Enhanced CD4^+^ T-cell frequencies could reflect a higher bacterial load during induction of the T-cell response. In our experience, particularly the magnitude of the CD4^+^ T-cell response correlates with the bacterial load (KL and H-WM, unpublished observation). However, we cannot exclude that IL-6Rα-deficient myeloid cells either provide stronger supportive signals or fail to deliver suppressive signals for the T-cell response.

Due to its intracellular replication, control of *L*. *monocytogenes* primarily relies on T_H_1 cells and cytotoxic CD8^+^ T cells. However, we also detected an increase in frequencies of T_H_17 cells in spleens of infected mice. Frequencies of IL-17A-secreting T_H_17 cells were reduced in *Il6*KO and *Il6ra*^fl/fl^×CD4^cre^ mice but not in sgp130FcTg mice indicating that in our model, development of T_H_17 cells was strictly dependent on classical IL-6 signaling which could not be compensated by IL-6 trans-signaling or IL-6 cluster-signaling.

In contrast to *Il6ra*^fl/fl^×CD4^cre^ mice, *Il6ra*^fl/fl^×LysM^cre^ mice showed impaired control of *L*. *monocytogenes*. Compared to the initial characterizations of *Il6*KO mice by Kopf et al. [6] and Dalrymple et al. [26], the increase in listeria titers in *Il6ra*^fl/fl^×LysM^cre^ mice was only modest. However, this result is consistent with our previous study in which *Il6*KO mice or mice treated with neutralizing anti-IL-6 mAb also show less pronounced increases in listeria titers than those observed in the original studies [10].

It is currently unclear why the deficient control of *L*. *monocytogenes* in *Il6ra*^fl/fl^×LysM^cre^ mice at early time points is restricted to the liver. Analysis of inflammatory cytokines and iNOS in livers of infected mice revealed only marginal differences between *Il6ra*^fl/fl^×LysM^cre^ and control mice. Neutrophils are recruited to spleen and liver in the early phase of listeria infection. Results on the requirement of neutrophils for the control of *L*. *monocytogenes* are conflicting but point to a role primarily in the liver [46, 47]. In our current study, infected *Il6ra*^fl/fl^×LysM^cre^ and control mice showed similar accumulation of neutrophils in spleen and liver, and cells only marginally differed in all phenotypically and functional analyses. Thus, there is so far no evidence for an impaired granulocyte response in *Il6ra*^fl/fl^×LysM^cre^ mice.

Early control of *L*. *monocytogenes* primarily depends on CCR2-mediated mobilization of inflammatory monocytes from the bone marrow and recruitment of these cells to spleen and liver [27, 28]. We observed normal accumulation of inflammatory monocytes in *Il6ra*^fl/fl^×LysM^cre^ mice, and migration of these cells towards CCL2 and fMLP was not inhibited. These results recapitulate our results from *Il6*KO mice, where we observed only minor alterations in inflammatory monocyte and granulocyte accumulation in listeria-infected tissues [10].

Following listeria infection, inflammatory monocytes migrate to sites of infection and mature to pro-inflammatory M1 macrophages. However, under specific conditions, such as the presence of type 2 cytokines, alternatively activated or M2 macrophages can develop. Alternatively activated macrophages participate in helminth infection and allergic inflammations but are also implicated in tissue repair and regeneration [48, 49]. Mauer and colleagues could show that IL-6 induces the expression of the IL-4Rα and thereby promotes development of alternatively activated macrophages [4]. In line with their results, we observed induction of IL-4Rα by IL-6 in BMDM, and inflammatory monocytes from infected *Il6ra*^fl/fl^×LysM^cre^ mice showed reduced IL-4Rα expression, indicating that they might be less sensitive to IL-4. In the liver, listeria rapidly infect and destroy Kupffer cells, which triggers the release of the alarmin IL-33 from hepatocytes. Interestingly, IL-33 induces the production of IL-4 in basophils and thereby promotes maturation of alternatively activated macrophages. After clearance of the infection, these macrophages replace the destroyed Kupffer cells and restore the integrity of the liver tissue [50]. Thus, IL-4 is available in the listeria-infected liver and can direct differentiation of IL-4Rα^+^ inflammatory monocytes. Phenotypic and functional characterization of inflammatory monocytes in infected *Il6ra*^fl/fl^×LysM^cre^ mice revealed a more activated phenotype and enhanced capability to produce TNFα, which would be consistent with an enhanced M1 polarization. However, surface expression of the M2 marker MMR1 was similar and the phagocytic activity was reduced when compared to inflammatory cells from infected control mice. Thus, alterations of IL-6Rα-deficient inflammatory monocytes can only partially be explained by a shift towards an M1 development.

CD38 has been described as an M1 macrophage marker [22] and we observed strong CD38 induction in BMDM cultured with IFNγ and LPS. However, CD38 was also induced by IL-4 and induction depended on IL-6 and IL-6Rα expression on BMDM. Interestingly, CD38 was significantly reduced on inflammatory monocytes from infected *Il6ra*^fl/fl^×LysM^cre^ mice suggesting that in our model, CD38 expression is mainly driven by IL-6. CD38 has enzymatic activity and can use nicotinamide adenine dinucleotide (NAD) and nicotinamide adenine dinucleotide phosphate (NADP) to generate adenosine diphosphoribose (ADPR), cyclic adenosine diphosphoribose (cADPR) or nicotinic acid adenine dinucleotide phosphate (NAADP). All three products act as second messengers and induce an increase in cytosolic Ca^2+^ [51, 52]. In neutrophils, monocytes and dendritic cells, CD38 cooperates with chemotactic receptors in the induction of Ca^2+^ signaling [53]. *Cd38*KO mice show an ameliorated course of disease in different autoimmune or immune pathology models as well as diminished pathogen control in infection models [52, 54–61], which in most models is associated with reduced accumulation and activation of granulocytes or macrophages. We could demonstrate that *Cd38*KO mice are highly susceptible to *L*. *monocytogenes* [25]. Infected *Cd38*KO mice present with reduced accumulation of neutrophils and inflammatory monocytes in the spleen, but enhanced recruitment of inflammatory monocytes to the liver [25]. Thus, impaired listeria control cannot simply be explained by altered recruitment of these cells. However, we observed diminished uptake of listeria by CD38-deficient macrophages, which is consistent with a recent report that CD38-deficient macrophages are impaired in phagocytosis [25, 32]. In our current study, CD38^-^ cells were less efficient in phagocytosis of beads. In the liver of infected *Il6ra*^fl/fl^×LysM^cre^ mice, phagocytosis was significantly reduced in both CD38^+^ and CD38^-^ cells. Thus, reduced phagocytosis together with diminished CD38 expression could be responsible for the impaired listeria control *Il6ra*^fl/fl^×LysM^cre^ mice. However, we can currently not exclude that induction of CD38 expression and control of phagocytosis are independent processes regulated by IL-6 in inflammatory monocytes.

Impaired control of *L*. *monocytogenes* infection might not be restricted to diminished bacteria uptake but also to defective restriction of intracellular replication, since we could recover higher numbers of listeria from IL-6Rα-deficient BMDM. Macrophages rely on the production of NO to kill intracellular listeria. However, NO production by BMDM was independent from IL-6 and IL-6Rα expression. Therefore, the cause of impaired listeria control in macrophages is currently unclear.

In conclusion, we demonstrate that IL-6-mediated protection against *L*. *monocytogenes* depends on classical IL-6 signaling in myeloid cells. Impaired IL-6 signaling is associated with alterations in the differentiation and function of these cells, which subsequently prevent effective control of bacteria.

## Supporting information

S1 Fig Gating strategies and IL-6Rα expression on cells from *Il6ra*^fl/fl^×CD4^cre^ and *Il6ra*^fl/fl^×LysM^cre^ mice. Leukocytes were first defined by granularity (SSC-A) and size (FSC-A). Cell doublets were excluded. Dead cells were excluded by Pacific Orange staining (PacO). (**A**) Myeloid cells were defined as CD11b^+^. Granulocytes were identified as Ly6C^int^ Gr1^hi^. From cells that were not defined as granulocytes, inflammatory monocytes were characterized as Ly6C^hi^ F4/80^int^. Ly6C^low^ monocytes were defined as Ly6C^int^ and F4/80^low^ and macrophages were defined Ly6C^low^ and F4/80^hi^. (**B**) After the exclusion of dead cells, T cells were defined as CD8^+^ or CD4^+^. Dendritic cells were defined as CD11c^+^ and MHC II^hi^. B cells were identified by CD19 expression. (**C**) CD4^+^ T cells, CD8^+^ T cells, B cells, dendritic cells, macrophages, granulocytes, inflammatory monocytes and Ly6C^low^ monocytes were stained for surface IL-6Rα to determine the efficacy of the cell-specific knockout. Concatamer plots combining indicated cells from different mouse lines are given. From left to right: isotype control, CD4^cre^, *Il6ra*^fl/fl^×CD4^cre^, *Il6ra*^fl/fl^ and *Il6ra*^fl/fl^×LysM^cre^. Numbers represent mean fluorescence intensity.
(PDF)

S2 Fig IL-6 signaling does not influence specific T-cell responses against *Listeria monocytogenes*. sgp130FcTg, *Il6*KO and WT mice were infected i.v. with 1×10^4^ listeria recombinant for ovalbumin. Eight days p.i., spleen cells were isolated and stimulated with LLO_189-201_ and OVA_257-264_ (**A, B**), or ionomycin and PMA (**C, D**). Intracellular IFNγ and IL-17A expression was determined by flow cytometry. Frequencies of IFNγ^+^ CD4^+^ and IFNγ^+^ CD8^+^ cells from spleens of infected sgp130FcTg (open bars in **A, C**), *Il6*KO (open bars in **B, D**) and wildtype control mice (filled bars in **A**–**D**) without (none) or with *in vitro* stimulation are depicted. Each group contained 4 to 6 mice. Mean ± SEM are shown. Significance was determined by unpaired student’s t-test. One representative experiment of two is shown.
(PDF)

S3 Fig Abrogation of classical IL-6 signaling in myeloid cells impairs listeria control and causes enhanced CD4^+^ T cell response. (A) *Il6ra*^fl/fl^×LysM^cre^ and *Il6ra*^fl/fl^ control mice were infected i.v. with 2×10^3^ listeria. Five days p.i., titers in liver and spleen were determined. Results for individually analyzed mice and median bars are shown. Significance was determined with the Mann-Whitney U test. Results from two independent experiments were pooled. (B) *Il6ra*^fl/fl^×LysM^cre^ and *Il6ra*^fl/fl^ control mice were infected i.v. with 1×10^4^ listeria recombinant for ovalbumin. Eight days p.i., spleen cells were isolated and stimulated with LLO_189-201_ and OVA_257-264_. Intracellular IFNγ expression was determined by flow cytometry. Frequencies of IFNγ^+^CD4^+^ or IFNγ^+^CD8^+^ spleen cells following *in vitro* peptide stimulation are shown. Bars represent mean ± SEM of 5 to 6 mice per group. Significance was determined with student’s t-test.
(PDF)

S4 Fig Induction of inflammatory cytokines in *Il6ra*^fl/fl^×LysM^cre^ mice infected with *Listeria monocytogenes*. *Il6ra*^fl/fl^×LysM^cre^ and *Il6ra*^fl/fl^ littermate control mice were infected i.v. with 2×10^4^ listeria and analyzed d2 p.i. Uninfected mice were included as additional controls. Protein lysates and RNA were isolated from spleen and liver. Cytokine expression in protein lysates of spleen (**A, B**) and liver (**C, D**). Expression of *Il1b* (**E**), *Nos2* (**F**) and *Il6* (**G**) mRNA was analyzed using qPCR and normalized to 18S RNA using the ΔCT method. Bars represent mean ± SEM and circles represent individually analyzed mice with dashes as medians. Significance was determined by 2way ANOVA.
(PDF)

S5 Fig Phenotype and cytokine expression of granulocytes and Ly6C^low^ monocytes. *Il6ra*^fl/fl^×LysM^cre^ and *Il6ra*^fl/fl^ littermate control mice were infected i.v. with 2×10^4^ listeria. Two days p.i., liver and spleen cells from infected mice were isolated and granulocytes (**A-D**) and Ly6C^low^ monocytes (**E-H**) were characterized by flow cytometry (gating strategy is given in S1A Fig). Mean fluorescence intensity (MFI) of different surface proteins of granulocytes (**A, B**) and Ly6C^low^ monocytes (**E, F**). Spleen cells were stimulated with LPS for 4 h. Frequency of TNFα^+^ and IL-6^+^ granulocytes (**C**) and Ly6C^low^ monocytes (**G**). MFI of intracellular TNFα and IL-6 of granulocytes (**D**) and Ly6C^low^ monocytes (**H**). One representative experiment of two with 5 to 7 mice per group is shown. Bars represent mean ± SEM. Significance was determined with student’s t-test.
(PDF)

S1 Table Antibodies used for this study. Specificity (antigen), conjugated fluorochrome, supplier, clone and ID of each monoclonal antibody used for flow cytometric analyses.
(PDF)

## Abbreviations

fMLP: N-formyl-met-leu-phe
Lm: *Listeria monocytogenes*
LmOVA: Ovalbumin-recombinant *Listeria monocytogenes* OVA
MMR1: Macrophage mannose receptor 1

## References

[1] Scheller J, Chalaris A, Schmidt-Arras D, Rose-John S. The pro- and anti-inflammatory properties of the cytokine interleukin-6. Biochim Biophys Acta. Elsevier B.V.; 2011;1813: 878–88. 10.1016/j.bbamcr.2011.01.034 21296109

[2] Liu F, Poursine-Laurent J, Wu HY, Link DC. Interleukin-6 and the granulocyte colony-stimulating factor receptor are major independent regulators of granulopoiesis in vivo but are not required for lineage commitment or terminal differentiation. Blood. 1997;90: 2583–90. Available: http://www.ncbi.nlm.nih.gov/pubmed/9326224 9326224

[3] Chomarat P, Banchereau J, Davoust J, Palucka AK. IL-6 switches the differentiation of monocytes from dendritic cells to macrophages. Nat Immunol. 2000;1: 510–4. 10.1038/82763 11101873

[4] Mauer J, Chaurasia B, Goldau J, Vogt MC, Ruud J, Nguyen KD, et al Signaling by IL-6 promotes alternative activation of macrophages to limit endotoxemia and obesity-associated resistance to insulin. Nat Immunol. 2014;15: 423–30. 10.1038/ni.2865 24681566 PMC4161471

[5] Luig M, Kluger M a, Goerke B, Meyer M, Nosko A, Yan I, et al Inflammation-Induced IL-6 Functions as a Natural Brake on Macrophages and Limits GN. J Am Soc Nephrol. 2015; 1–11. 10.1681/ASN.2014060620 25655068 PMC4483592

[6] Kopf M, Baumann H, Freer G, Freudenberg M, Lamers M, Kishimoto T, et al Impaired immune and acute-phase responses in interleukin-6-deficient mice. Nature. 1994;368: 339–42. 10.1038/368339a0 8127368

[7] Nurieva RI, Chung Y, Hwang D, Yang XO, Kang HS, Ma L, et al Generation of T Follicular Helper Cells Is Mediated by Interleukin-21 but Independent of T Helper 1, 2, or 17 Cell Lineages. Immunity. 2008;29: 138–149. 10.1016/j.immuni.2008.05.009 18599325 PMC2556461

[8] Bettelli E, Carrier Y, Gao W, Korn T, Strom TB, Oukka M, et al Reciprocal developmental pathways for the generation of pathogenic effector TH17 and regulatory T cells. Nature. 2006;441: 235–8. 10.1038/nature04753 16648838

[9] Lauder SN, Jones E, Smart K, Bloom A, Williams AS, Hindley JP, et al Interleukin-6 limits influenza-induced inflammation and protects against fatal lung pathology. Eur J Immunol. 2013;43: 2613–25. 10.1002/eji.201243018 23857287 PMC3886386

[10] Hoge J, Yan I, Jänner N, Schumacher V, Chalaris A, Steinmetz OM, et al IL-6 controls the innate immune response against Listeria monocytogenes via classical IL-6 signaling. J Immunol. 2013;190: 703–11. 10.4049/jimmunol.1201044 23241882

[11] Smith KA, Maizels RM. IL-6 controls susceptibility to helminth infection by impeding Th2 responsiveness and altering the Treg phenotype in vivo. Eur J Immunol. 2014;44: 150–61. 10.1002/eji.201343746 24185641 PMC3992848

[12] Fishman D, Faulds G, Jeffery R, Mohamed-Ali V, Yudkin JS, Humphries S, et al The effect of novel polymorphisms in the interleukin-6 (IL-6) gene on IL-6 transcription and plasma IL-6 levels, and an association with systemic-onset juvenile chronic arthritis. J Clin Invest. 1998;102: 1369–76. 10.1172/JCI2629 9769329 PMC508984

[13] Yoshizaki K, Matsuda T, Nishimoto N, Kuritani T, Taeho L, Aozasa K, et al Pathogenic significance of interleukin-6 (IL-6/BSF-2) in Castleman’s disease. Blood. 1989;74: 1360–7. Available: http://www.ncbi.nlm.nih.gov/pubmed/2788466 2788466

[14] Boulanger MJ, Chow D, Brevnova EE, Garcia KC. Hexameric structure and assembly of the interleukin-6/IL-6 alpha-receptor/gp130 complex. Science. 2003;300: 2101–4. 10.1126/science.1083901 12829785

[15] Schöbitz B, Pezeshki G, Pohl T, Hemmann U, Heinrich PC, Holsboer F, et al Soluble interleukin-6 (IL-6) receptor augments central effects of IL-6 in vivo. FASEB J. 1995;9: 659–64. Available: http://www.ncbi.nlm.nih.gov/pubmed/7768358 7768358 10.1096/fasebj.9.8.7768358

[16] Oberg H-H, Wesch D, Grüssel S, Rose-John S, Kabelitz D. Differential expression of CD126 and CD130 mediates different STAT-3 phosphorylation in CD4+CD25- and CD25high regulatory T cells. Int Immunol. 2006;18: 555–63. 10.1093/intimm/dxh396 16540526

[17] Rose-John S, Heinrich PC. Soluble receptors for cytokines and growth factors: generation and biological function. Biochem J. 1994;300 (Pt 2): 281–90. Available: http://www.ncbi.nlm.nih.gov/pubmed/8002928 8002928 10.1042/bj3000281 PMC1138158

[18] Jostock T, Müllberg J, Ozbek S, Atreya R, Blinn G, Voltz N, et al Soluble gp130 is the natural inhibitor of soluble interleukin-6 receptor transsignaling responses. Eur J Biochem. 2001;268: 160–7. Available: http://www.ncbi.nlm.nih.gov/pubmed/11121117 11121117 10.1046/j.1432-1327.2001.01867.x

[19] Heink S, Yogev N, Garbers C, Herwerth M, Aly L, Gasperi C, et al Trans-presentation of IL-6 by dendritic cells is required for the priming of pathogenic TH17 cells. Nat Immunol. 2017;18: 74–85. 10.1038/ni.3632 27893700 PMC5164931

[20] Ramaswamy V, Cresence VM, Rejitha JS, Lekshmi MU, Dharsana KS, Prasad SP, et al Listeria—review of epidemiology and pathogenesis. J Microbiol Immunol Infect. 2007;40: 4–13. Available: http://eutils.ncbi.nlm.nih.gov/entrez/eutils/elink.fcgi?dbfrom=pubmed&id=17332901&retmode=ref&cmd=prlinks%5Cnpapers3://publication/uuid/DC8883CB-85F6-4753-B69F-4361696D511C 17332901

[21] Pamer EG. Immune responses to Listeria monocytogenes. Nat Rev Immunol. 2004;4: 812–23. 10.1038/nri1461 15459672

[22] Jablonski KA, Amici SA, Webb LM, Ruiz-Rosado J de D, Popovich PG, Partida-Sanchez S, et al Novel Markers to Delineate Murine M1 and M2 Macrophages. PLoS One. 2015;10: e0145342 10.1371/journal.pone.0145342 26699615 PMC4689374

[23] Wunderlich FT, Ströhle P, Könner AC, Gruber S, Tovar S, Brönneke HS, et al Interleukin-6 signaling in liver-parenchymal cells suppresses hepatic inflammation and improves systemic insulin action. Cell Metab. 2010;12: 237–49. 10.1016/j.cmet.2010.06.011 20816090

[24] Rabe B, Chalaris A, May U, Waetzig GH, Seegert D, Williams AS, et al Transgenic blockade of interleukin 6 transsignaling abrogates inflammation. Blood. 2008;111: 1021–8. 10.1182/blood-2007-07-102137 17989316

[25] Lischke T, Heesch K, Schumacher V, Schneider M, Haag F, Koch-Nolte F, et al CD38 controls the innate immune response against Listeria monocytogenes. Infect Immun. 2013;81: 4091–9. 10.1128/IAI.00340-13 23980105 PMC3811837

[26] Dalrymple SA, Lucian LA, Slattery R, McNeil T, Aud DM, Fuchino S, et al Interleukin-6-deficient mice are highly susceptible to Listeria monocytogenes infection: correlation with inefficient neutrophilia. Infect Immun. 1995;63: 2262–8. Available: http://www.ncbi.nlm.nih.gov/pubmed/7768607 7768607 10.1128/iai.63.6.2262-2268.1995 PMC173295

[27] Kurihara T, Warr G, Loy J, Bravo R. Defects in macrophage recruitment and host defense in mice lacking the CCR2 chemokine receptor. J Exp Med. 1997;186: 1757–62. 10.1084/jem.186.10.1757 9362535 PMC2199145

[28] Jia T, Serbina N V, Brandl K, Zhong MX, Leiner IM, Charo IF, et al Additive roles for MCP-1 and MCP-3 in CCR2-mediated recruitment of inflammatory monocytes during Listeria monocytogenes infection. J Immunol. 2008;180: 6846–6853. 10.4049/jimmunol.180.10.6846 18453605 PMC2386263

[29] Lavigne MC, Murphy PM, Leto TL, Gao JL. The N-formylpeptide receptor (FPR) and a second Gi-coupled receptor mediate fMet-Leu-Phe-stimulated activation of NADPH oxidase in murine neutrophils. Cell Immunol. 2002;218: 7–12. 10.1016/S0008-8749(02)00564-6 12470609

[30] Liu M, Chen K, Yoshimura T, Liu Y, Gong W, Wang A, et al Formylpeptide receptors are critical for rapid neutrophil mobilization in host defense against Listeria monocytogenes. Sci Rep. 2012;2: 786 10.1038/srep00786 23139859 PMC3493074

[31] Yang J, Zhang L, Yu C, Yang X-F, Wang H. Monocyte and macrophage differentiation: circulation inflammatory monocyte as biomarker for inflammatory diseases. Biomark Res. 2014;2: 1 10.1186/2050-7771-2-1 24398220 PMC3892095

[32] Kang J, Park K-HH, Kim J-JJ, Jo E-KK, Han M-KK, Kim U-HH. The role of CD38 in Fcγ receptor (FcγR)-mediated phagocytosis in murine macrophages. J Biol Chem. 2012;287: 14502–14. 10.1074/jbc.M111.329003 22396532 PMC3340275

[33] Rincon M. Interleukin-6: from an inflammatory marker to a target for inflammatory diseases. Trends Immunol. 2012;33: 571–7. 10.1016/j.it.2012.07.003 22883707

[34] Tanaka T, Katada Y, Higa S, Fujiwara H, Wang W, Saeki Y, et al Enhancement of T helper2 response in the absence of interleukin (IL-)6; an inhibition of IL-4-mediated T helper2 cell differentiation by IL-6. Cytokine. 2001;13: 193–201. 10.1006/cyto.2000.0828 11237426

[35] Romani L, Mencacci A, Cenci E, Spaccapelo R, Toniatti C, Puccetti P, et al Impaired neutrophil response and CD4+ T helper cell 1 development in interleukin 6-deficient mice infected with Candida albicans. J Exp Med. 1996;183: 1345–55. 10.1084/jem.183.4.1345 8666893 PMC2192497

[36] Nish SA, Schenten D, Wunderlich T, Pope SD, Gao Y, Hoshi N, et al T cell-intrinsic role of IL-6 signaling in primary and memory responses. Elife. 2014;2014: 1–21. 10.7554/eLife.01949 24842874 PMC4046568

[37] Rincón M, Anguita J, Nakamura T, Fikrig E, Flavell RA. Interleukin (IL)-6 directs the differentiation of IL-4-producing CD4+ T cells. J Exp Med. 1997;185: 461–9. 10.1084/jem.185.3.461 9053446 PMC2196041

[38] Diehl S, Anguita J, Hoffmeyer A, Zapton T, Ihle JN, Fikrig E, et al Inhibition of Th1 differentiation by IL-6 is mediated by SOCS1. Immunity. 2000;13: 805–815. 10.1016/S1074-7613(00)00078-9 11163196

[39] Ladel CH, Blum C, Dreher A, Reifenberg K, Kopf M, Kaufmann SHE. Lethal tuberculosis in interleukin-6-deficient mutant mice. Infect Immun. 1997;65: 4843–4849. Available: http://www.ncbi.nlm.nih.gov/pubmed/9353074 9353074 10.1128/iai.65.11.4843-4849.1997 PMC175695

[40] Longhi MP, Wright K, Lauder SN, Nowell MA, Jones GW, Godkin AJ, et al Interleukin-6 is crucial for recall of influenza-specific memory CD4 + T cells. PLoS Pathog. 2008;4: 2–9. 10.1371/journal.ppat.1000006 18389078 PMC2279258

[41] La Flamme AC, Pearce EJ. The absence of IL-6 does not affect Th2 cell development in vivo, but does lead to impaired proliferation, IL-2 receptor expression, and B cell responses. J Immunol. 1999;162: 5829–5837. Available: http://www.ncbi.nlm.nih.gov/pubmed/10229817 10229817

[42] MacLeod MKL, McKee AS, David A, Wang J, Mason R, Kappler JW, et al Vaccine adjuvants aluminum and monophosphoryl lipid A provide distinct signals to generate protective cytotoxic memory CD8 T cells. Proc Natl Acad Sci U S A. 2011;108: 7914–9. 10.1073/pnas.1104588108 21518876 PMC3093483

[43] Böttcher JP, Schanz O, Garbers C, Zaremba A, Hegenbarth S, Kurts C, et al IL-6 trans-Signaling-Dependent Rapid Development of Cytotoxic CD8+ T Cell Function. Cell Rep. 2014;8: 1318–1327. 10.1016/j.celrep.2014.07.008 25199826

[44] Yan I, Schwarz J, Lücke K, Schumacher N, Schumacher V, Schmidt S, et al ADAM17 controls IL-6 signaling by cleavage of the murine IL-6Rα from the cell surface of leukocytes during inflammatory responses. J Leukoc Biol. 2016;99: 749–60. 10.1189/jlb.3A0515-207R 26561568

[45] Waetzig GH, Rose-John S. Hitting a complex target: an update on interleukin-6 trans-signalling. Expert Opin Ther Targets. 2012;16: 225–36. 10.1517/14728222.2012.660307 22324903

[46] Shi C, Hohl TM, Leiner I, Equinda MJ, Fan X, Pamer EG. Ly6G+ neutrophils are dispensable for defense against systemic Listeria monocytogenes infection. J Immunol. 2011;187: 5293–8. 10.4049/jimmunol.1101721 21976773 PMC3208088

[47] Carr KD, Sieve AN, Indramohan M, Break TJ, Lee S, Berg RE. Specific depletion reveals a novel role for neutrophil-mediated protection in the liver during Listeria monocytogenes infection. Eur J Immunol. 2011;41: 2666–2676. 10.1002/eji.201041363 21660934 PMC3517125

[48] Martinez FO, Gordon S. The M1 and M2 paradigm of macrophage activation: time for reassessment. F1000Prime Rep. 2014;6: 1–13. 24669294 10.12703/P6-13 PMC3944738

[49] Van Dyken SJ, Locksley RM. Interleukin-4- and Interleukin-13-Mediated Alternatively Activated Macrophages: Roles in Homeostasis and Disease. Annu Rev Immunol. 2013;31: 317–343. 10.1146/annurev-immunol-032712-095906 23298208 PMC3606684

[50] Blériot C, Dupuis T, Jouvion G, Eberl G, Disson O, Lecuit M. Liver-resident macrophage necroptosis orchestrates type 1 microbicidal inflammation and type-2-mediated tissue repair during bacterial infection. Immunity. 2015;42: 145–58. 10.1016/j.immuni.2014.12.020 25577440

[51] Guse AH. Calcium mobilizing second messengers derived from NAD. Biochim Biophys Acta. Elsevier B.V.; 2015;1854: 1132–1137. 10.1016/j.bbapap.2014.12.015 25534250

[52] Partida-Sánchez S, Cockayne D a, Monard S, Jacobson EL, Oppenheimer N, Garvy B, et al Cyclic ADP-ribose production by CD38 regulates intracellular calcium release, extracellular calcium influx and chemotaxis in neutrophils and is required for bacterial clearance in vivo. Nat Med. 2001;7: 1209–16. 10.1038/nm1101-1209 11689885

[53] Partida-Sánchez S, Iribarren P, Moreno-García ME, Gao J-L, Murphy PM, Oppenheimer N, et al Chemotaxis and calcium responses of phagocytes to formyl peptide receptor ligands is differentially regulated by cyclic ADP ribose. J Immunol. 2004;172: 1896–906. 10.4049/jimmunol.172.3.1896 14734775

[54] Postigo J, Iglesias M, Cerezo-Wallis D, Rosal-Vela A, García-Rodríguez S, Zubiaur M, et al Mice deficient in CD38 develop an attenuated form of collagen type II-induced arthritis. PLoS One. 2012;7: 1–9. 10.1371/journal.pone.0033534 22438945 PMC3306406

[55] Choe C un, Lardong K, Gelderblom M, Ludewig P, Leypoldt F, Koch-Nolte F, et al CD38 exacerbates focal cytokine production, postischemic inflammation and brain injury after focal cerebral ischemia. PLoS One. 2011;6: 1–8. 10.1371/journal.pone.0019046 21625615 PMC3097994

[56] Gally F, Hartney JM, Janssen WJ, Perraud A-L. CD38 plays a dual role in allergen-induced airway hyperresponsiveness. Am J Respir Cell Mol Biol. 2009;40: 433–42. 10.1165/rcmb.2007-0392OC 18931329 PMC2720120

[57] Schneider M, Schumacher V, Lischke T, Lücke K, Meyer-Schwesinger C, Velden J, et al CD38 is expressed on inflammatory cells of the intestine and promotes intestinal inflammation. PLoS One. 2015;10: 1–16. 10.1371/journal.pone.0126007 25938500 PMC4418770

[58] Partida-Sánchez S, Randall TD, Lund FE. Innate immunity is regulated by CD38, an ecto-enzyme with ADP-ribosyl cyclase activity. Microbes Infect. 2003;5: 49–58. 10.1016/S1286-4579(02)00055-2 12593973

[59] Viegas MS, Silva T, Monteiro MM, do Carmo A, Martins TC. Knocking out of CD38 accelerates development of a lupus-like disease in lpr mice. Rheumatology (Oxford). 2011;50: 1569–77. 10.1093/rheumatology/ker178 21586522

[60] Estrada-Figueroa LA, Ramírez-Jiménez Y, Osorio-Trujillo C, Shibayama M, Navarro-García F, García-Tovar C, et al Absence of CD38 delays arrival of neutrophils to the liver and innate immune response development during hepatic amoebiasis by Entamoeba histolytica. Parasite Immunol. 2011;33: 661–668. 10.1111/j.1365-3024.2011.01333.x 21919917

[61] Cervantes-Sandoval I, Serrano-Luna J de J, García-Latorre E, Tsutsumi V, Shibayama M. Characterization of brain inflammation during primary amoebic meningoencephalitis. Parasitol Int. 2008;57: 307–13. 10.1016/j.parint.2008.01.006 18374627

