# Supplementary figures and images for: Control of Listeria monocytogenes infection requires classical IL-6 signaling in myeloid cells
Source: PLoS One. 2018 Aug 31;13(8):e0203395. doi: 10.1371/journal.pone.0203395 (PMC6118394; doi:10.1371/journal.pone.0203395)

Figure S1

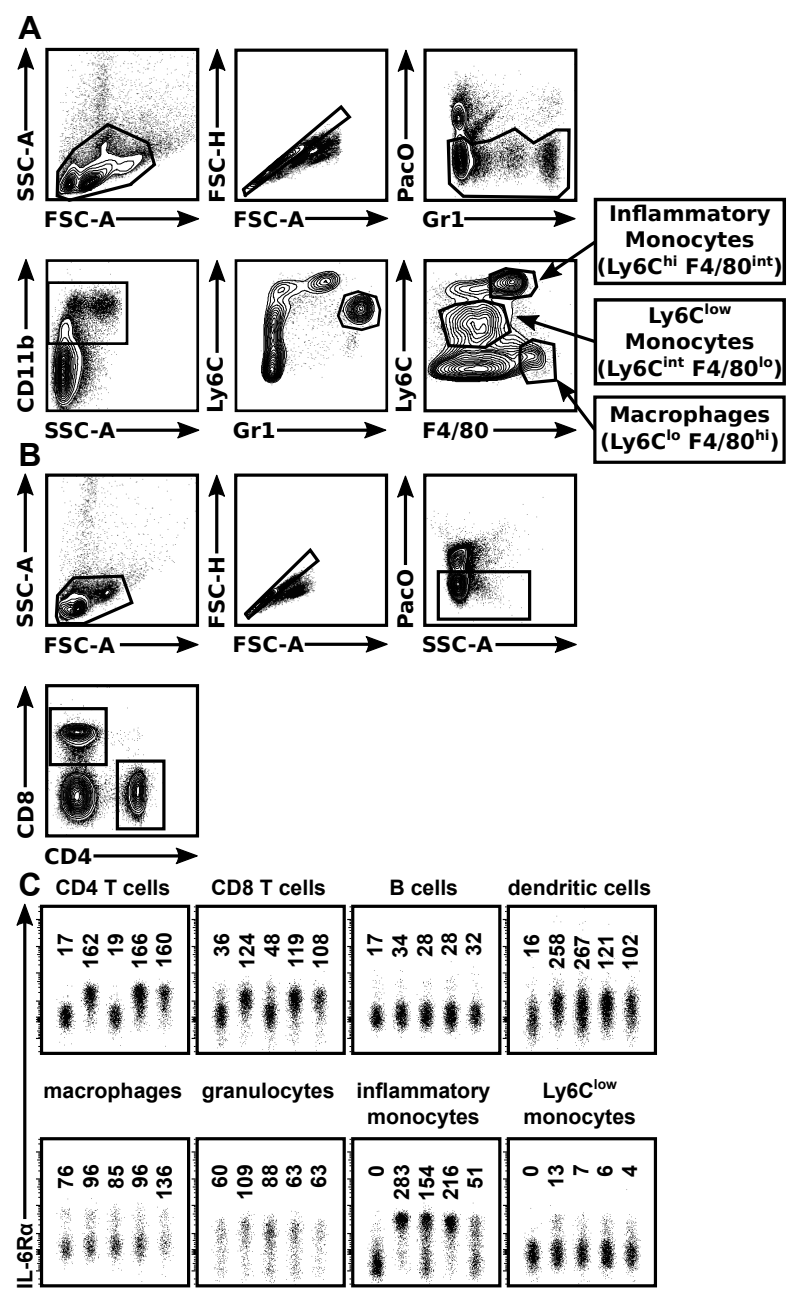

Supplement: S1 Fig — Leukocytes were first defined by granularity (SSC-A) and size (FSC-A). Cell doublets were excluded. Dead cells were excluded by Pacific Orange staining (PacO). (A) Myeloid cells were defined as CD11b+. Granulocytes were identified as Ly6Cint Gr1hi. From cells that were not defined as granulocytes, inflammatory monocytes were characterized as Ly6Chi F4/80int. Ly6Clow monocytes were defined as Ly6Cint and F4/80low and macrophages were defined Ly6Clow and F4/80hi. (B) After the exclusion of dead cells, T cells were defined as CD8+ or CD4+. Dendritic cells were defined as CD11c+ and MHC IIhi. B cells were identified by CD19 expression. (C) CD4+ T cells, CD8+ T cells, B cells, dendritic cells, macrophages, granulocytes, inflammatory monocytes and Ly6Clow monocytes were stained for surface IL-6Rα to determine the efficacy of the cell-specific knockout. Concatamer plots combining indicated cells from different mouse lines are given. From left to right: isotype control, CD4cre, Il6rafl/fl×CD4cre, Il6rafl/fl and Il6rafl/fl×LysMcre. Numbers represent mean fluorescence intensity. (PDF) [file pone.0203395.s001.pdf]

Figure S2

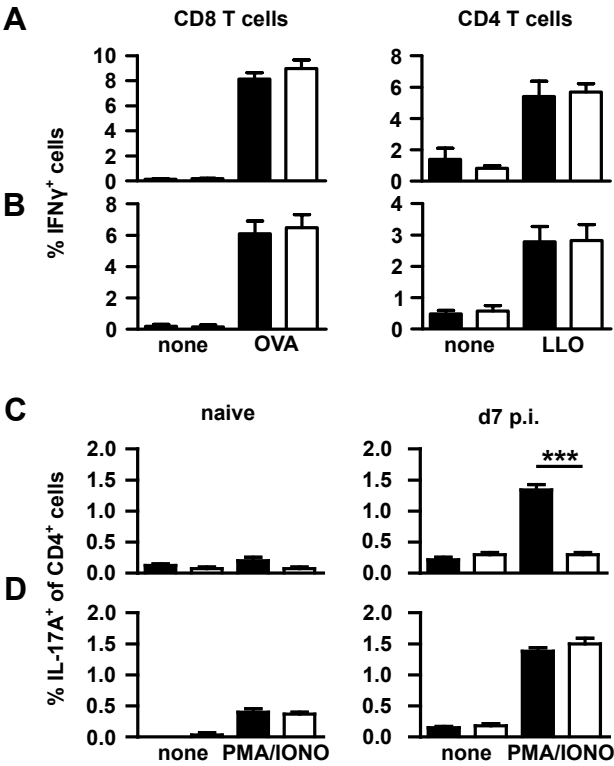

Supplement: S2 Fig — sgp130FcTg, Il6KO and WT mice were infected i.v. with 1×10^4^ listeria recombinant for ovalbumin. Eight days p.i., spleen cells were isolated and stimulated with LLO189-201 and OVA257-264 (A, B), or ionomycin and PMA (C, D). Intracellular IFNγ and IL-17A expression was determined by flow cytometry. Frequencies of IFNγ+ CD4+ and IFNγ+ CD8+ cells from spleens of infected sgp130FcTg (open bars in A, C), Il6KO (open bars in B, D) and wildtype control mice (filled bars in A–D) without (none) or with in vitro stimulation are depicted. Each group contained 4 to 6 mice. Mean ± SEM are shown. Significance was determined by unpaired student’s t-test. One representative experiment of two is shown. (PDF) [file pone.0203395.s002.pdf]

Figure S3

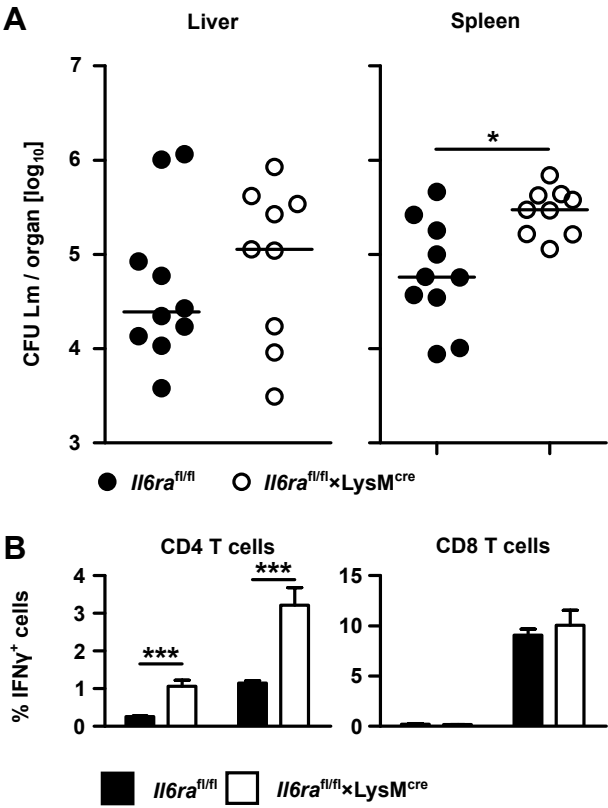

Supplement: S3 Fig — (A) Il6rafl/fl×LysMcre and Il6rafl/fl control mice were infected i.v. with 2×10^3^ listeria. Five days p.i., titers in liver and spleen were determined. Results for individually analyzed mice and median bars are shown. Significance was determined with the Mann-Whitney U test. Results from two independent experiments were pooled. (B) Il6rafl/fl×LysMcre and Il6rafl/fl control mice were infected i.v. with 1×10^4^ listeria recombinant for ovalbumin. Eight days p.i., spleen cells were isolated and stimulated with LLO189-201 and OVA257-264. Intracellular IFNγ expression was determined by flow cytometry. Frequencies of IFNγ+CD4+ or IFNγ+CD8+ spleen cells following in vitro peptide stimulation are shown. Bars represent mean ± SEM of 5 to 6 mice per group. Significance was determined with student’s t-test. (PDF) [file pone.0203395.s003.pdf]

Figure S4

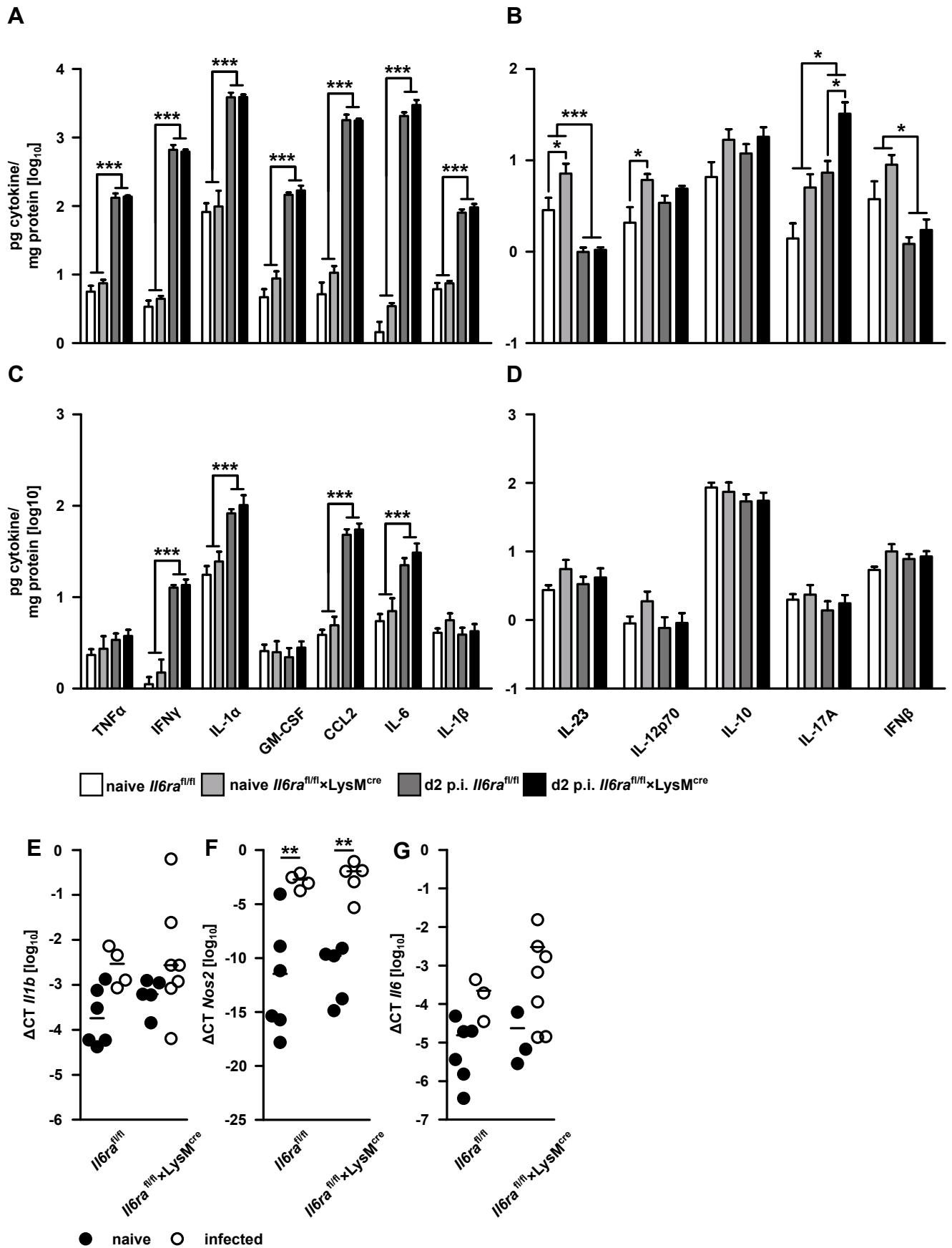

Supplement: S4 Fig — Il6rafl/fl×LysMcre and Il6rafl/fl littermate control mice were infected i.v. with 2×10^4^ listeria and analyzed d2 p.i. Uninfected mice were included as additional controls. Protein lysates and RNA were isolated from spleen and liver. Cytokine expression in protein lysates of spleen (A, B) and liver (C, D). Expression of Il1b (E), Nos2 (F) and Il6 (G) mRNA was analyzed using qPCR and normalized to 18S RNA using the ΔCT method. Bars represent mean ± SEM and circles represent individually analyzed mice with dashes as medians. Significance was determined by 2way ANOVA. (PDF) [file pone.0203395.s004.pdf]

Figure S5

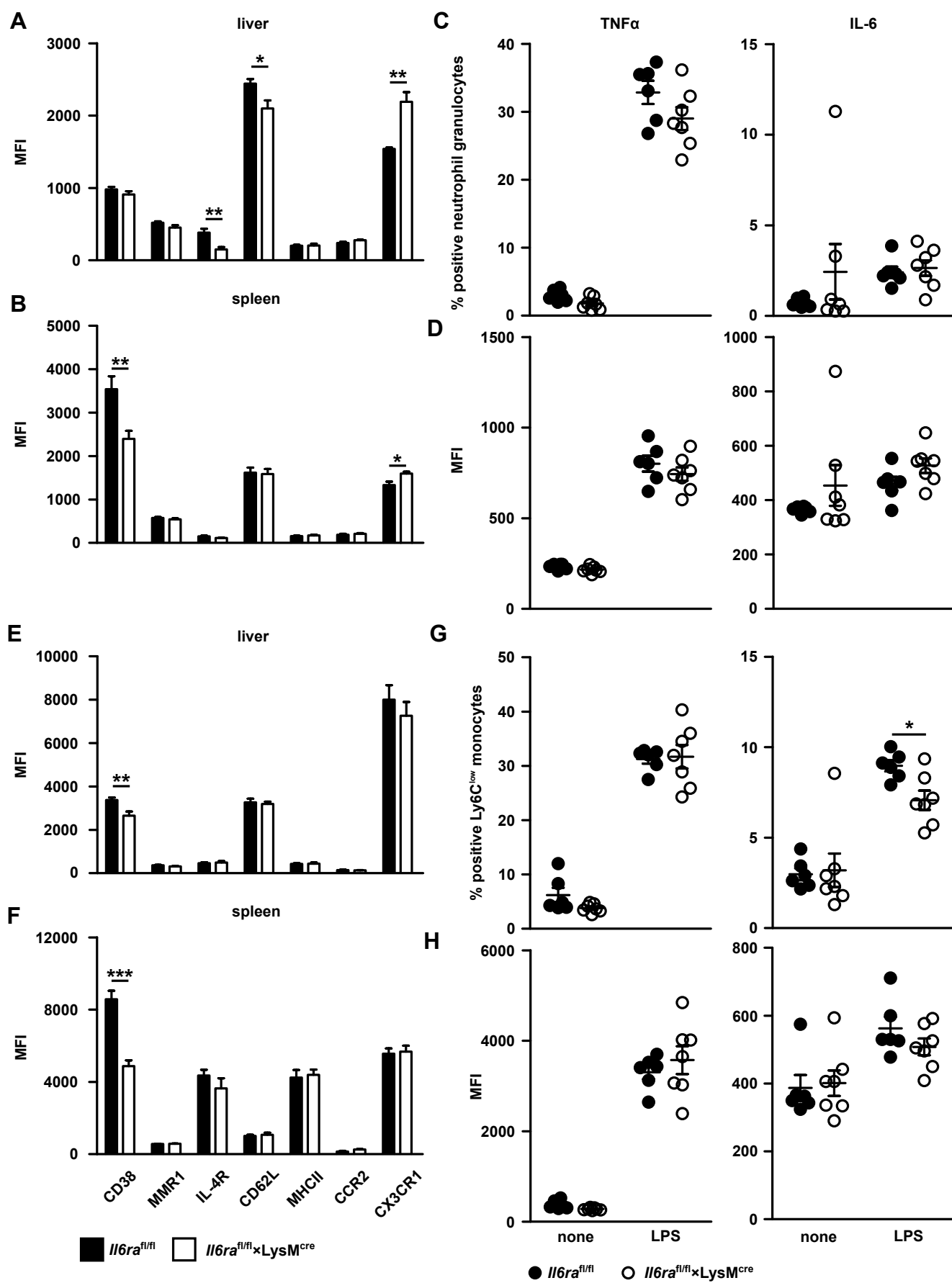

Supplement: S5 Fig — Il6rafl/fl×LysMcre and Il6rafl/fl littermate control mice were infected i.v. with 2×10^4^ listeria. Two days p.i., liver and spleen cells from infected mice were isolated and granulocytes (A-D) and Ly6Clow monocytes (E-H) were characterized by flow cytometry (gating strategy is given in S1A Fig). Mean fluorescence intensity (MFI) of different surface proteins of granulocytes (A, B) and Ly6Clow monocytes (E, F). Spleen cells were stimulated with LPS for 4 h. Frequency of TNFα+ and IL-6+ granulocytes (C) and Ly6Clow monocytes (G). MFI of intracellular TNFα and IL-6 of granulocytes (D) and Ly6Clow monocytes (H). One representative experiment of two with 5 to 7 mice per group is shown. Bars represent mean ± SEM. Significance was determined with student’s t-test. (PDF) [file pone.0203395.s005.pdf]
