# Supplementary material for: Control of Listeria monocytogenes infection requires classical IL-6 signaling in myeloid cells
Source: PLoS One. 2018 Aug 31;13(8):e0203395. doi: 10.1371/journal.pone.0203395 (PMC6118394; doi:10.1371/journal.pone.0203395)
Supplement: S1 Table — Specificity (antigen), conjugated fluorochrome, supplier, clone and ID of each monoclonal antibody used for flow cytometric analyses. (PDF) [file pone.0203395.s006.pdf]

Table S1

Antigen	Fluorochrome	Supplier	Clone	Antibody ID
CCR2	AF700	R&D Systems	FAB5538N	AB_2725739
CD11b	PerCP	BioLegend	M1/70	AB_2129374
CD11b	PE-Cy7	BioLegend	M1/70	AB_312799
CD11c	BV650	Becton Dickinson	HL3	AB_2725779
CD19	PerCP-Cy5.5	eBiosciences	eBio1D3	AB_906215
CD206	PerCP-Cy5.5	BioLegend	C068C2	AB_2561992
CD38	PE-Cy7	BioLegend	90	AB_2275531
CD4	AF700	BioLegend	RM4-5	AB_493699
CD4	APC-Cy7	BioLegend	RM4-5	AB_312727
CD4	BV785	BioLegend	RM4-5	AB_2563053
CD44	PE-Cy7	BioLegend	IM7	AB_830787
CD62L	APC-Cy7	BioLegend	MEL-14	AB_830787
CD8	PerCP	BioLegend	53-6.7	AB_893423
CD8	BV510	BioLegend	53-6.7	AB_2563057
CX3CR1	PE	BioLegend	SA011F11	AB_2564315
F4/80	BV421	BioLegend	BM8	AB_11203717
F4/80	AF647	BioLegend	BM8	AB_893480
GR1	PacB	BioLegend	RB6-8C5	AB_893556
Gr1	FITC	BioLegend	RB6-8C5	AB_313371
IFNg	AF647	eBiosciences	XMG1.2	AB_10393003
IL-17A	PE	eBiosciences	eBio17B7	AB_763582
IL-1bpro	APC	eBiosciences	NJTEN3	AB_10670739
IL-4R	PE	BioLegend	I015F8	AB_2561730
IL-6	PE	BioLegend	MP5-20F3	AB_315338
IL-6R	PE	BioLegend	D7715A7	AB_313677
Ly6C	AF647	BioLegend	HK1.4	AB_1236550
Ly6C	FITC	Becton Dickinson	AL-21	AB_394628
Ly6C	APC-Cy7	BioLegend	HK1.4	AB_10640120
MHCII	AF700	BioLegend	M5/114.15.2	AB_493727
TNFa	PE-Cy7	BioLegend	MP6-XT22	AB_2256076
